# Genomic epidemiology of multidrug-resistant and ESBL-producing *Proteus mirabilis* in a military referral hospital in Ethiopia

**DOI:** 10.1371/journal.pone.0355508

**Published:** 2026-08-11

**Authors:** Yacob Mohammed Yusuf, Adey Feleke Desta, Desta Ayana, Helen Nigussie Aychegrew, Rajiha Abubeker, Biruk Samson, Tolewak Sura, Atsbeha Gebreegziabxier, Abebe Asseffa Negeri, Chala Bashea, Melak Getu, Gebremedhin Gebremichael, Amete Mihret, Eleni Kidane, Ebisa Mezgebu, Sami Sebri, Gemechu Tadesse, Getachew Tollera, Abraham Ali, Gurja Belay Woldemichael

**Affiliations:** 1 Armed Forces Comprehensive Specialized Hospital, Addis Ababa, Ethiopia; 2 Department of Microbial Sciences and Genetics, College of Natural and Computational Sciences, Addis Ababa University, Addis Ababa, Ethiopia; 3 Ethiopian Public Health Institute, Addis Ababa, Ethiopia; 4 Animal Health Institute, Oromia, Ethiopia; 5 Widassie Diagnostic Centre, Addis Ababa, Ethiopia; Zhejiang University, CHINA

## Abstract

Multidrug-resistant *Proteus mirabilis* has emerged as an important healthcare-associated pathogen; however, genomic epidemiological data from sub-Saharan Africa remain limited, particularly in military hospital settings. This study aimed to characterize the phenotypic and genomic resistance profiles of *P. mirabilis* isolates recovered from a military referral hospital in Ethiopia between May 2024 and May 2025. A total of 99 non-duplicate clinical isolates were analyzed using antimicrobial susceptibility testing according to Clinical and Laboratory Standards Institute (CLSI) guidelines, with phenotypic detection of extended-spectrum β-lactamase (ESBL) and carbapenemase production. Whole-genome sequencing was performed on a selected subset of 25 representative ESBL- and/or carbapenemase-associated *P. mirabilis* isolates to identify resistance genes, determine multilocus sequence types (MLST), analyze plasmid replicons, and reconstruct core-genome phylogeny. Using phenotypic detection, 96.0% of isolates were found to be resistant to at least one third- or fourth-generation cephalosporin, and 99.0% were classified as multidrug-resistant. ESBL production was confirmed in 60.6% (60/99) of isolates, while 6.1% (6/99) demonstrated carbapenemase activity. Genomic analysis revealed diverse ESBL determinants dominated by *bla*_PER-13_ (40.0%) and *bla*_VEB-6_ (28.0%), with additional detection of *bla*_CTX-M-65_. MLST analysis identified nine sequence types, with ST135 predominating (32.0%). Phylogenetic analysis demonstrated moderate genetic heterogeneity, with evidence of multiple lineages circulating within the hospital setting; however, transmission dynamics could not be definitively determined. Plasmid analysis showed a structured replicon distribution dominated by IncQ1 (36.0%) and several Col-type plasmids. These findings demonstrate extensive multidrug resistance associated with diverse ESBL determinants and multiple plasmid replicon types among *P. mirabilis* isolates in a military healthcare setting. The results highlight the need for strengthened antimicrobial stewardship, improved infection prevention strategies, and integration of genomic surveillance in comparable healthcare environments.

## 1 Introduction

Antimicrobial resistance (AMR) is a major global public health threat, contributing substantially to morbidity and mortality worldwide. Recent analyses indicate that bacterial AMR has been associated with at least one million deaths annually since 1990, with projections suggesting more than 39 million deaths globally between now and 2050. The burden remains disproportionately high in low- and middle-income countries [1,2]. The increasing prevalence of multidrug-resistant Gram-negative bacteria has further limited therapeutic options and complicated infection management in healthcare settings [2].

*Proteus mirabilis* is an opportunistic Gram-negative pathogen commonly associated with urinary tract infections, wound infections, and healthcare-associated infections. It is particularly known for its role in complicated urinary tract infections and catheter-associated infections due to its swarming motility, biofilm formation, and urease production [3,4]. Although historically considered less critical than other Enterobacterales, *P. mirabilis* has increasingly been implicated in multidrug-resistant infections [4,5].

The emergence of extended-spectrum β-lactamase (ESBL)-producing Enterobacterales has significantly reduced the efficacy of third-generation cephalosporins. While CTX-M enzymes dominate globally, other ESBL families, including PER and VEB variants, have been reported in various regions [6,7]. In addition, carbapenem resistance among Enterobacterales is of particular concern, as carbapenems are often reserved as last-line agents for severe infections [2,8]. Although carbapenem resistance is more frequently described in *Klebsiella pneumoniae* and *Escherichia coli*, recent studies have reported the emergence of carbapenem non-susceptible *P. mirabilis* [9,10].

Whole-genome sequencing (WGS) has become an important tool for characterizing antimicrobial resistance determinants, plasmid replicons, and clonal lineages. Compared with phenotypic testing alone, WGS enables detailed analysis of resistance gene distribution, mobile genetic elements, and strain relatedness, thereby improving epidemiological understanding of resistant pathogens in healthcare settings [11,12].

Despite the increasing global importance of multidrug-resistant *P. mirabilis*, genomic epidemiological data from military healthcare settings in sub-Saharan Africa remain scarce. This study addresses this knowledge gap by integrating phenotypic resistance characterization with whole-genome sequencing, phylogenetic analysis, virulence profiling, and plasmid replicon analysis of clinical *P. mirabilis* isolates recovered from a military referral hospital in Ethiopia. This study aimed to determine the phenotypic and genomic characteristics of multidrug-resistant *P. mirabilis* isolates recovered from a military referral hospital in Ethiopia, with particular emphasis on ESBL and carbapenem resistance, resistance gene distribution, plasmid replicons, virulence determinants and clonal structure.

## 2 Materials and methods

### 2.1 Study setting

This study was conducted at the Armed Forces Comprehensive Specialized Hospital (AFCSH) in Addis Ababa, Ethiopia. AFCSH is a tertiary referral hospital operating under the Federal Ministry of Defense and provides healthcare services primarily to active military personnel, retired members, civilian employees, and military families. The hospital has approximately 913 inpatient beds and multidisciplinary clinical wards.

### 2.2 Study design and study period

A hospital-based cross-sectional laboratory study was conducted from May 2024 to May 2025. Routine clinical specimens submitted for culture and antimicrobial susceptibility testing as part of standard patient care were prospectively processed.

### 2.3 Clinical specimens and isolate selection

Clinical specimens obtained from patients clinically suspected of bacterial infection, for whom attending physicians requested culture and antimicrobial susceptibility testing, were included in this study. Specimen types comprised urine, wound/pus and blood samples processed at the Armed Forces Comprehensive Specialized Hospital.

During the study period, a total of 99 non-duplicate *P. mirabilis* isolates were recovered from clinical specimens processed at the hospital. All isolates with complete laboratory records and interpretable antimicrobial susceptibility testing results were included in the final phenotypic analysis, while isolates with incomplete laboratory information or uninterpretable results were excluded.

Among the included isolates, those demonstrating resistance to at least one third- or fourth-generation cephalosporin (ceftriaxone, cefotaxime, ceftazidime, or cefepime) and/or reduced susceptibility to carbapenems were further evaluated for phenotypic detection of ESBL production and carbapenemase activity. Based on these phenotypic results, 25 representative *Proteus mirabilis* isolates enriched for ESBL-producing and/or carbapenemase-producing phenotypes were purposively selected for whole-genome sequencing to capture diversity in specimen type, ward/source, multidrug resistance profile, and antimicrobial resistance characteristics. A comparison of the sequenced subset with the complete isolate collection is provided in S1 Table.

All eligible non-duplicate *P. mirabilis* isolates recovered during the study period were included in the analysis. No a priori sample size calculation was performed, as this was a laboratory-based surveillance study including all available isolates within the defined timeframe.

### 2.4 Bacterial identification

Initial identification was performed using Gram staining and conventional biochemical tests including urease production, indole reaction, citrate utilization, triple sugar iron agar reactions, hydrogen sulfide production, and motility testing. Species confirmation was performed using matrix-assisted laser desorption/ionization time-of-flight mass spectrometry (MALDI-TOF MS) according to the manufacturer’s instructions.

### 2.5 Antimicrobial susceptibility testing

Antimicrobial susceptibility testing was performed using the Kirby–Bauer disk diffusion method on Mueller–Hinton agar according to the Clinical and Laboratory Standards Institute (CLSI) M100, 33rd edition guidelines [13]. The antimicrobial agents tested were ceftriaxone (30 µg), cefotaxime (30 µg), ceftazidime (30 µg), cefepime (30 µg), aztreonam (30 µg), imipenem (10 µg), meropenem (10 µg), ciprofloxacin (5 µg), trimethoprim–sulfamethoxazole (1.25/23.75 µg), amikacin (30 µg), piperacillin–tazobactam (100/10 µg), and gentamicin (10 µg). Zone diameters were interpreted according to CLSI M100, 33rd edition criteria. Isolates categorized as intermediate or resistant were considered non-susceptible for comparative analyses. *Escherichia coli* ATCC 25922 was used as the quality control strain. Plates were incubated aerobically at 35 ± 2°C for 16–18 hours before interpretation. For comparative analyses, isolates categorized as intermediate or resistant were grouped and reported as non-susceptible.

Multidrug-resistant (MDR) was defined as non-susceptibility to at least one agent in three or more antimicrobial classes according to the international consensus criteria proposed by Magiorakos et al. [14].

### 2.6 Phenotypic detection of ESBL and carbapenemase production

Screening for ESBL production was performed on isolates showing reduced susceptibility to one or more third- or fourth-generation cephalosporins, including ceftriaxone, cefotaxime, ceftazidime, and cefepime. Confirmation of ESBL production was performed using the double-disk synergy test (DDST) according to CLSI M100 (33rd edition) recommendations. Briefly, an amoxicillin–clavulanic acid (20/10 µg) disk was placed at the center of a Mueller–Hinton agar plate, and cefotaxime (30 µg), ceftazidime (30 µg), ceftriaxone (30 µg), and cefepime (30 µg) disks were placed at an appropriate distance around the central disk. Enhancement of the inhibition zone toward the amoxicillin–clavulanic acid disk (keyhole effect) was interpreted as confirmation of ESBL production. Carbapenemase production was evaluated using the modified carbapenem inactivation method (mCIM) according to CLSI M100 (33rd edition) guidelines. Isolates showing reduced susceptibility to meropenem and/or imipenem were further tested by mCIM. For mCIM testing, a 10 µg meropenem disk was incubated in a suspension of the test isolate and subsequently placed on a Mueller–Hinton agar plate inoculated with *Escherichia coli* ATCC 25922. Following incubation, inhibition zone diameters were interpreted according to CLSI criteria, and isolates meeting the CLSI-defined thresholds were classified as carbapenemase producers.

### 2.7 DNA Extraction and whole-genome sequencing

Genomic DNA was extracted using the QIAamp DNA Mini Kit (QIAGEN, Hilden, Germany) according to the manufacturer’s protocol. Sequencing libraries were prepared using the Illumina DNA Prep Kit and sequenced on the Illumina NextSeq 550 platform with paired-end 2 × 150 bp reads at the Integrated Genomics and Bioinformatics for Research and Surveillance Facility of the Ethiopian Public Health Institute.

### 2.8 Bioinformatics analysis

Raw reads quality was assessed using FastQC (v0.12.11) to assess read quality metrics [15]. Adapter sequences and low-quality bases were trimmed using fastp [16]. De novo genome assembly was performed using Shovill, which internally employs SKESA (v1.1.0) for assembly. Assembly quality metrics, including total genome size, GC content, the number of contigs, N50 values, and genome completeness were assessed using QUAST (v5.0.2) [17], and genome annotation was conducted using Prokka [18] and genome completeness was assessed using BUSCO (v5.7.1) against the bacteria_odb10 lineage dataset. Antimicrobial resistance genes were identified using AMRFinderPlus (v3.12.8; database version 2024-05-02.2) [19]. In silico multilocus sequence typing (MLST) was performed using the PubMLST scheme [20]. Core-genome single-nucleotide variants (SNVs) were identified using Snippy (v4.6.0) with the *Proteus mirabilis* reference genome N002952. A maximum-likelihood phylogenetic tree was constructed using IQ-TREE 2 (v2.2.0) under the best-fit substitution model selected by ModelFinder, with 1,000 ultrafast bootstrap replicates to assess branch support [21]. The resulting phylogenetic tree was visualized and midpoint-rooted using Interactive Tree of Life (iTOL). All bioinformatics analyses were performed using default parameters unless otherwise specified. Virulence-associated genes were identified using ABRicate with the Virulence Factor Database (VFDB) under default parameters. Assembly quality statistics, including genome size, BUSCO completeness, N50 values, contig counts, and GC content, were compiled for all sequenced isolates and are provided in S3 Table.

### 2.9 Statistical analysis

Descriptive statistics were used to summarize antimicrobial resistance patterns and genomic characteristics. Associations between categorical variables were evaluated using Chi-square or Fisher’s exact tests, as appropriate. A two-tailed p-value < 0.05 was considered statistically significant. Statistical analyses were performed using SPSS version 25.0 (IBM Corp., Armonk, NY, USA). Given the small number of carbapenemase-producing isolates, analyses involving carbapenemase-positive and carbapenemase-negative groups were considered exploratory and interpreted with caution. Because several contingency tables contained sparse cells, particularly for comparisons involving carbapenemase-positive isolates, odds ratios and corresponding 95% confidence intervals were not reported because the resulting effect-size estimates would be unstable. Accordingly, Fisher's exact test was used to assess associations, and comparisons involving carbapenemase-positive isolates should be interpreted as exploratory.

### 2.10 Ethics statement

Ethical approval for this study was obtained from the Institutional Review Board of the College of Natural and Computational Sciences, Addis Ababa University (CNS-IRB; Ref. No. CNSCDO/707/16/2024). Permission to conduct the study was also granted by the management of the Armed Forces Comprehensive Specialized Hospital. Clinical specimens were collected as part of routine diagnostic care. The study involved laboratory analysis of bacterial isolates only and did not include direct patient contact. All data were anonymized prior to analysis, and no personal identifiers were recorded or used. The requirement for informed consent was waived by the ethics committee due to the use of de-identified clinical samples.

## 3 Results

### 3.1 Demographic and isolate selection

A total of 99 non-duplicate *P. mirabilis* isolates were recovered from routine clinical specimens at the AFCSH. Urine was the main specimen source, accounting for 52 (52.5%) isolates, followed by wound samples 42 (42.4%) isolates and blood samples 5 (5.1%) isolates. Most isolates were obtained from young adult patients, with the highest proportion in the 18–24-year age group (58/99; 58.6%), followed by those aged 25–34 years (27/99; 27.3%). In contrast, fewer isolates were recovered from older age groups, including 35–44 years (6/99; 6.1%), 45–54 years (5/99; 5.1%), and 55–64 years (1/99; 1.0%), with only a small number of patients aged ≥65 years (2/99; 2.0%). The majority of isolates were recovered from military personnel (89/99; 89.9%), while smaller proportions were obtained from retired individuals (5/99; 5.1%), civilian patients (3/99; 3.0%), and military family members (2/99; 2.0%). Male patients contributed 95 isolates (96.0%), whereas female patients accounted for 4 (4.0%) isolates. The surgical wards contributed the largest proportion of isolates (38/99; 38.4%), followed by the outpatient department (30/99; 30.3%) and medical wards (21/99; 21.2%). Smaller proportions were obtained from orthopedic wards (7/99; 7.1%) and the intensive care unit (3/99; 3.0%) (Table 1).

**Table 1:** Demographic and Specimen types characteristics of patients contributing *Proteus mirabilis* isolates at the Armed Forces Comprehensive Specialized Hospital (AFCSH), Addis Ababa (n = 99).

Characteristic	Category	No. of Patients	Percentage (%)
Sex	Male	95	96.0
	Female	4	4.0
Age group	18–24 years	58	58.6
	25–34 years	27	27.3
	35–44 years	6	6.1
	45–54 years	5	5.1
	55–64 years	1	1.0
	≥65 years	2	2.0
Population type	Military	89	89.9
	Military family	2	2.0
	Civil	3	3.0
	Retired	5	5.1
Specimen type	Urine	52	52.5
	Wound	42	42.4
	Blood	5	5.1
Ward type	OPD Medical	30 21	30.3 21.2
	Surgical	38	38.4
	Orthopedics	7	7.1
	ICU	3	3.0

### 3.2 Phenotypic detection of ESBL and carbapenemase-producing *Proteus mirabilis*

Of the 99 *Proteus mirabilis* isolates, 95 (96.0%) were resistant to at least one third- or fourth-generation cephalosporin (ceftriaxone, cefotaxime, ceftazidime, or cefepime), indicating widespread reduced susceptibility to extended-spectrum cephalosporins. These isolates were subjected to phenotypic ESBL screening using the double-disk synergy method, which confirmed ESBL production in 60 isolates, corresponding to 63.2% of screened isolates and 60.6% of the total isolates. In addition, 6 (6.1%) isolates were positive for carbapenemase production as determined by the modified carbapenem inactivation method (mCIM).

### 3.3 Antimicrobial Resistance patterns of ESBL-positive and ESBL-negative *Proteus mirabilis* isolates

ESBL-producing isolates (n = 60) generally showed higher resistance to third- and fourth-generation cephalosporins and aztreonam compared with ESBL-negative isolates (n = 39). Resistance to gentamicin was significantly higher among ESBL-producing isolates (p = 0.038).

No statistically significant differences were observed for other antibiotics (p > 0.05) (Table 2).

**Table 2:** Comparison of antimicrobial resistance between ESBL-producing and non-producing *P. mirabilis* isolates.

Antibiotic	ESBL+ (n = 60) n (%)	ESBL− (n = 39) n (%)	p-value
Ceftriaxone (CRO)	50 (83.3%)	27 (69.2%)	0.138
Cefotaxime (CTX)	54 (90.0%)	29 (74.4%)	0.052
Ceftazidime (CAZ)	49 (81.7%)	26 (66.7%)	0.099
Cefepime (FEP)	43 (71.7%)	24 (61.5%)	0.380
Aztreonam (ATM)	50 (83.3%)	26 (66.7%)	0.087
Imipenem (IMI)	15 (25.0%)	10 (25.6%)	1.000
Meropenem (MRO)	12 (20.0%)	9 (23.1%)	0.803
Ciprofloxacin (CIP)	46 (76.7%)	32 (82.1%)	0.619
Trimethoprim–sulfamethoxazole (TS)	48 (80.0%)	30 (76.9%)	0.803
Amikacin (AK)	4 (6.7%)	4 (10.3%)	0.708
Piperacillin–tazobactam (TPZ)	38 (63.3%)	21 (53.8%)	0.404
Gentamicin (CN)	32 (53.3%)	12 (30.8%)	0.038*

Statistically significant (p < 0.05). Percentages represent non-susceptible isolates (intermediate + resistant). P-values were calculated using Fisher’s exact test.

### 3.4 Antimicrobial resistance pattern of carbapenemase-positive and -negative *Proteus mirabilis* isolates

Carbapenemase-producing isolates (n = 6) showed significantly higher resistance to imipenem and meropenem compared with carbapenemase-negative isolates (n = 93) (p < 0.001 for both). Resistance to gentamicin was also significantly higher among carbapenemase-producing isolates (p = 0.006). No statistically significant differences were observed for other antibiotics (p > 0.05) (Table 3).

**Table 3:** Comparison of antimicrobial resistance between carbapenemase-producing and non-producing *P. mirabilis* isolates.

Antibiotic	Carbapenemase-positive (n = 6) n (%)	Carbapenemase-negative (n = 93) n (%)	p-value
Ceftriaxone (CRO)	6 (100.0%)	71 (76.3%)	0.333
Cefotaxime (CTX)	5 (83.3%)	78 (83.9%)	1.000
Ceftazidime (CAZ)	5 (83.3%)	70 (75.3%)	1.000
Cefepime (FEP)	5 (83.3%)	62 (66.7%)	0.661
Aztreonam (ATM)	5 (83.3%)	71 (76.3%)	1.000
Imipenem (IMI)	6 (100.0%)	19 (20.4%)	<0.001*
Meropenem (MRO)	6 (100.0%)	15 (16.1%)	<0.001*
Ciprofloxacin (CIP)	6 (100.0%)	72 (77.4%)	0.337
Trimethoprim–sulfamethoxazole (TS)	5 (83.3%)	73 (78.5%)	1.000
Amikacin (AK)	1 (16.7%)	7 (7.5%)	0.405
Piperacillin–tazobactam (TPZ)	6 (100.0%)	53 (57.0%)	0.078
Gentamicin (CN)	6 (100.0%)	38 (40.9%)	0.006*

Statistically significant (p < 0.05). Percentages represent non-susceptible isolates (intermediate + resistant). P-values were calculated using Fisher’s exact test.

### 3.5 Multidrug-resistant profiles

**Fig 1:**
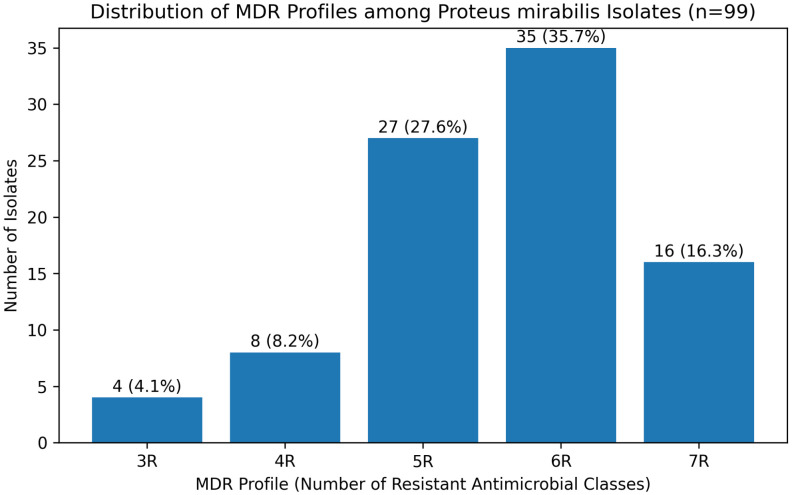
Distribution of multidrug-resistant (MDR) profiles among Proteus mirabilis isolates (n = 99). MDR profiles are expressed as the number of antimicrobial classes to which each isolate was resistant (3R–7R). The most common resistance profiles were 6R (35.7%) and 5R (27.6%), indicating widespread accumulation of resistance across multiple antimicrobial classes.

Multidrug resistance was widespread among the isolates. Ninety-eight (99.0%) *P. mirabilis* strains were resistant to three or more (≥3) antimicrobial classes, fulfilling the definition of multidrug-resistant (MDR) [14]. Only one isolate showed limited resistance, being resistant to two antimicrobial classes. Among MDR isolates, the extent of resistance ranged from 3R to 7R (i.e., resistance to 3–7 antimicrobial classes). The most frequent resistance profiles were 6R and 5R. The distribution of MDR categories is shown in Fig 1.

### 3.6 Phenotypic–genotypic concordance of ESBL detection

Among the 25 whole-genome sequenced Proteus mirabilis isolates, 20 were phenotypically confirmed as ESBL producers by the modified double-disk synergy test (MDDST), of which 17 (85.0%) harbored at least one ESBL-associated gene identified by whole-genome sequencing. The detected ESBL determinants included bla_PER-13_, bla_VEB-6_, bla_CTX-M-65_, and bla_VEB-5_, with bla_PER-13_ and bla_VEB-6_ being the most frequently detected. The concordance between phenotypic ESBL detection and whole-genome sequencing is summarized in Table 4.

**Table 4:** Phenotypic–genotypic concordance of ESBL detection among whole-genome sequenced Proteus mirabilis isolates (n = 25).

Part A. 2 × 2 concordance table			
Phenotypic ESBL (MDDST)	ESBL gene detected	No ESBL gene detected	Total
Positive	17	3	20
Negative	5	0	5
Total	22	3	25
**Part B**. **Diagnostic performance**			
**Parameter**		**Value**	
Sensitivity		77.3% (17/22)	
Specificity		0.0% (0/3)	
Positive predictive value (PPV)		85.0% (17/20)	
Negative predictive value (NPV)		0.0% (0/5)	
Overall agreement		68.0% (17/25)	

**Footnote:** Whole-genome sequencing detection of ESBL-associated genes (blaPER-13, blaVEB-6, blaCTX-M-65, and blaVEB-5) was used as the reference method. ESBL phenotype was determined using the modified double-disk synergy test (MDDST).

Three phenotypically ESBL-positive isolates (EPHIPM007, EPHIPM008, and EPHIPM013) did not carry recognized ESBL-associated genes. Two of these isolates harbored only bla_TEM-1_, a narrow-spectrum β-lactamase not typically associated with resistance to extended-spectrum cephalosporins, whereas one isolate lacked detectable acquired β-lactamase genes. Conversely, five isolates (EPHIPM001, EPHIPM006, EPHIPM019, EPHIPM021, and EPHIPM025) carried ESBL-associated genes but were not classified as ESBL producers by phenotypic testing, demonstrating discordance between phenotypic and genotypic ESBL detection.

Using whole-genome sequencing as the reference method, phenotypic ESBL detection demonstrated a sensitivity of 77.3%, specificity of 0.0%, positive predictive value of 85.0%, negative predictive value of 0.0%, and an overall agreement of 68.0%. These findings indicate partial concordance between phenotypic and genotypic ESBL detection. Because only three isolates lacked ESBL-associated genes, the specificity and negative predictive value should be interpreted cautiously. Overall, the observed discordance highlights the complementary value of combining phenotypic testing with whole-genome sequencing for accurate characterization of ESBL-producing *P. mirabilis* isolates.

### 3.7 Genomic characterization of sequenced isolates

The quality of genome assemblies was assessed using standard assembly metrics. Assembly sizes ranged from 3.81 Mb to 4.67 Mb, with GC contents ranging from 38.69% to 39.31%. BUSCO completeness values were high across all isolates (98.6%–99.6%), indicating near-complete genome assemblies. The number of contigs ranged from 45 to 424, while N50 values ranged from 107,403 bp to 218,681 bp. Overall, these metrics indicate that the assemblies were of sufficient quality for downstream analyses, including antimicrobial resistance gene detection, multilocus sequence typing, virulence profiling, plasmid replicon analysis, and phylogenetic reconstruction (S3 Table).

#### 3.7.1 Multilocus sequence typing (MLST) analysis.

MLST analysis revealed 9 distinct sequence types among the 25 sequenced isolates, indicating moderate genetic heterogeneity. ST135 was the dominant lineage (32.0%), followed by ST287 and ST178 (each 16.0%). The remaining isolates were distributed across six additional STs, each representing ≤8% of the collection. This distribution suggests both clonal expansion of selected lineages and parallel circulation of multiple genetically distinct strains within the hospital environment. Assembly quality metrics have been provided in S3 Table and summarized in the Results section.

#### 3.7.2 Core-genome phylogeny and genomic epidemiology.

Core-genome phylogenetic analysis demonstrated moderate genetic heterogeneity among the sequenced isolates, with three principal clades largely corresponding to ST135, ST178, and ST287 lineages. ST135 formed the largest phylogenetic cluster, suggesting partial clonal expansion within the hospital environment. However, the presence of additional distinct clusters indicates concurrent circulation of multiple lineages rather than a single outbreak strain.

The distribution of ESBL determinants across wards further supports endemic circulation. The predominant *bla*_PER-13_ gene was detected across surgical, medical, orthopedic, and intensive care units, without restriction to a single ward. In contrast, *bla*_VEB-6_ showed partial concentration in the outpatient department, while *bla*_CTX-M-65_ was sporadically distributed across different wards. These patterns are consistent with possible circulation of resistance determinants across different hospital units; however, additional epidemiological and genomic data are required to confirm transmission pathways.

**Fig 2:**
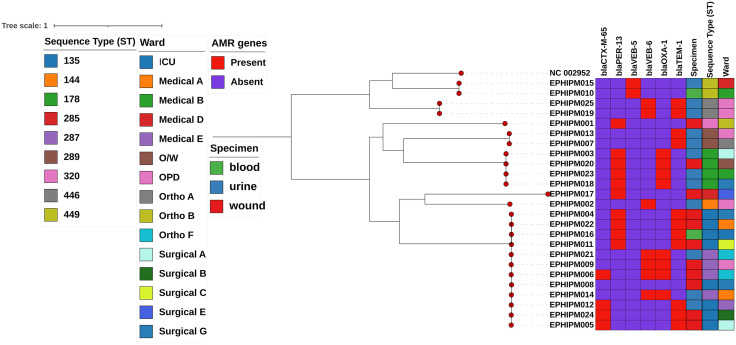
Core-genome maximum-likelihood phylogenetic tree of 25 ESBL- and carbapenemase-producing *P. mirabilis* isolates. The tree was constructed using SNV analysis against reference strain HI4320 (NC_002952) with IQ-TREE 2 (1,000 bootstrap replicates). The scale bar indicates nucleotide substitutions per site. Major clusters correspond primarily to ST135, ST178, and ST287 lineages. Red dots represent individual isolates.

Overall, the combined phylogenetic and ward-level analysis is consistent with possible circulation of MDR *P. mirabilis* lineages within the hospital setting; however, the selected sequencing subset and absence of epidemiological linkage data limit definitive conclusions regarding transmission dynamics. The phylogenetic relationships among the sequenced isolates and the distribution of major sequence types are shown in Fig 2.

### 3.8 β-lactamase and ESBL genes

Whole-genome sequencing revealed a structured distribution of β-lactamase genes among the sequenced *P. mirabilis* isolates.

Extended-spectrum β-lactamase (ESBL) genes were detected in 22 of 25 isolates (88.0%). The most prevalent ESBL determinant was bla_PER-13_, identified in 40.0% (10/25) of isolates. This was followed by bla_VEB-6_ (28.0%; 7/25), bla_CTX-M-65_ (4/25;16.0%), and bla_VEB-5_ (2/25;8.0%). These findings demonstrate the predominance of PER- and VEB-type ESBLs within this collection.

In addition to ESBL determinants, narrow-spectrum β-lactamases were frequently identified. The most common non-ESBL gene was bla_TEM-1_ (11/25;44.0%), followed by bla_OXA-1_ (8/25;32.0%). Although these enzymes are not typically associated with third- or fourth-generation cephalosporin hydrolysis, their co-occurrence with ESBL genes may contribute to broader β-lactam resistance phenotypes. No acquired carbapenemase genes were identified among the sequenced isolates, including those that were phenotypically positive for carbapenemase production by the modified carbapenem inactivation method (mCIM). Therefore, carbapenemase production detected by mCIM was not supported by the whole-genome sequencing analysis in the sequenced isolates.

Co-carriage of ESBL and non-ESBL β-lactamase genes was common, indicating accumulation of multiple resistance determinants within individual isolates. A complete gene presence/absence matrix is provided in S2 Table. The distribution of ESBL and non-ESBL β-lactamase genes among the sequenced isolates is summarized in Table 5.

**Table 5:** Distribution of ESBL and non-ESBL β-lactamase genes among sequenced *Proteus mirabilis* isolates (n = 25).

β-Lactamase Category	Gene	No. of Isolates	Percentage (%)
ESBL genes	*blaPER-13*	10	40.0
	*blaVEB-6*	7	28.0
	*blaCTX-M-65*	4	16.0
	*blaVEB-5*	2	8.0
Non-ESBL genes	*blaTEM-1*	11	44.0
	*blaOXA-1*	8	32.0

### 3.9 Non-β-lactam antibiotic resistance genes

Whole-genome sequencing identified a diverse array of resistance determinants conferring resistance to quinolones, aminoglycosides, macrolides, tetracyclines, trimethoprim, sulfonamides, and phenicols among the 25 sequenced *P. mirabilis* isolates.

Quinolone resistance was primarily mediated by plasmid-mediated quinolone resistance (PMQR) genes. The most prevalent determinant was *qnrD1* (7/25;28.0%), followed by aac(6′)-Ib-cr5 (4/25;16.0%). Additional PMQR genes, including qnrD2, qnrA1, and qnrD3, were each detected in 4.0% (1/25) of isolates.

Aminoglycoside resistance genes were widely distributed. The most common determinant was *aadA1* (80.0%; 20/25). Other frequently detected aminoglycoside-modifying enzyme genes included aph(6)-Id (48.0%), aph(3″)-Ib (48.0%), aadA5 (44.0%), and ant(2″)-Ia (44.0%), indicating extensive dissemination of aminoglycoside resistance mechanisms.

Macrolide and lincosamide resistance were mainly associated with mph(E) and lnu(F) (40.0% each), while ere(A) was detected in 32.0% of isolates.

Tetracycline resistance was universal and predominantly mediated by *tet(J)*, detected in all sequenced isolates (100%). The additional tetracycline resistance gene tet(A) was identified in 24.0% (6/25) of isolates.

Sulfonamide resistance genes were highly prevalent, with sul1 detected in 84.0% (21/25) and sul2 in 48.0% (12/25) of isolates. Trimethoprim resistance was primarily mediated by dfrA1 (18/25;72.0%) and dfrA17 (11/25;44.0%), while dfrA5 (16.0%) and dfrA15 (4.0%) were less frequently observed.

Among phenicol resistance determinants, catA was nearly universal (24/25;96.0%), followed by catA1 (16/25;64.0%) and catB3 (5/25;20.0%).

In addition to antibiotic resistance determinants, genes associated with heavy metal and biocide resistance were widely distributed. Tellurium resistance genes (terD and terZ) were present in all isolates (100%). Biocide resistance genes (qacEΔ1, 60.0%; qacE, 32.0%) and mercury resistance genes of the mer operon (44.0%) were also detected, suggesting potential co-selection of antimicrobial resistance through linked mobile genetic elements.

### 3.10 Virulence gene profile of sequenced *Proteus mirabilis* isolates

Virulence gene profiling of the 25 sequenced *Proteus mirabilis* isolates revealed a predominance of determinants associated with motility and cell surface structure. The lipopolysaccharide biosynthesis genes *gmhA*/*lpcA* were detected in all isolates (100%), indicating a highly conserved role in cell envelope integrity. Similarly, the flagellar regulatory gene *flhC* and the flagellar export component *fliN* were universally present, underscoring the importance of motility in the pathogenic potential of these isolates.

In contrast, the structural flagellar gene *flgG* was detected in 44% of isolates, while the flagellar protein gene *fliP* was identified in 16%. Genes associated with more specialized virulence mechanisms were less frequent, including the type VI secretion system-associated gene *vgrG1a* and the biofilm-related regulator *algQ*, each detected in only 4% of isolates.

**Fig 3:**
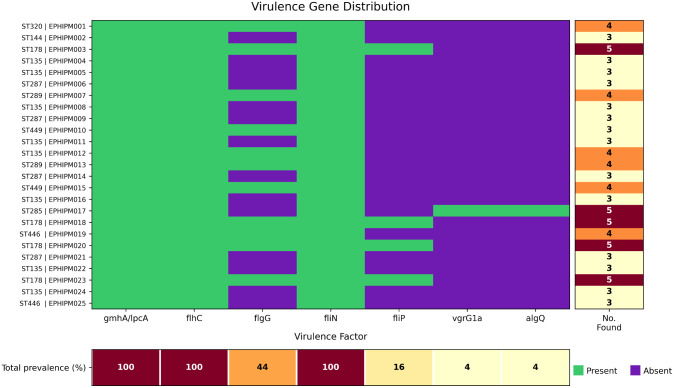
Heatmap showing the presence and absence of selected virulence-associated genes among 25 sequenced *P. mirabilis* isolates. Green indicates the presence of a virulence gene, whereas purple indicates its absence. Isolates are labelled according to sequence type (ST) and isolate identifier. The right-hand annotation track shows the total number of virulence genes detected in each isolate, while the bottom annotation track indicates the prevalence (%) of each virulence gene across all isolates. The figure demonstrates the widespread distribution of core virulence genes, including gmhA/IpcA, flhC, and fliN, whereas genes such as vgrG1a and algQ were detected in only a small proportion of isolates.

Overall, the distribution of virulence determinants suggests that core virulence features in this collection are dominated by conserved motility and cell surface-associated mechanisms, whereas advanced virulence systems are relatively uncommon. The number of virulence genes per isolate ranged from low to moderate, with variability observed across sequence types (Fig 3).

### 3.11 Plasmid replicon profiles of ESBL- and carbapenemase-producing *Proteus mirabilis*

Plasmid replicon analysis revealed that the sequenced *P. mirabilis* isolates carried a limited but structured plasmid repertoire. The most prevalent replicon was IncQ1, detected in 36.0% (9/25) of isolates. Small Col-type plasmids, including Col3M and Col(BS512), were also frequently identified and often co-occurred with IncQ1 within the same isolates.

Additional replicons—Col440I, Col156, Col (MG828), Col440II, ColKP3, and ColpVC were detected at lower frequencies, indicating a diverse but low-abundance plasmid population. Overall, the distribution of replicon types demonstrates the presence of multiple mobile genetic elements among ESBL- and carbapenemase-producing isolates in this cohort (Table 6).

**Table 6:** Distribution of plasmid replicon types among sequenced *Proteus mirabilis* isolates (n = 25).

Plasmid replicon	No. of isolates	Percentage (%)
IncQ1	9	36.0
Col3M	7	28.0
Col (BS512)	5	20.0
Col440I	2	8.0
Col156	1	4.0
Col (MG828)	1	4.0
Col440II	1	4.0
ColKP3	1	4.0
ColpVC	1	4.0

## 4 Discussion

This study provides a comprehensive phenotypic and genomic characterization of multidrug-resistant *Proteus mirabilis* isolates recovered from a military referral hospital in Ethiopia. The findings demonstrate a high burden of antimicrobial resistance, substantial diversity of ESBL determinants, and evidence of multiple multidrug-resistant lineages and plasmid replicon types within this unique healthcare setting.

The demographic distribution of isolates reflects the institutional structure of the hospital, with a predominance of young adult male patients and active-duty military personnel. This pattern likely represents the service population rather than a biological predisposition to infection. Similar trends have been reported in military and male-dominated healthcare settings [5,22]. The predominance of urine and wound specimens is consistent with the established epidemiology of *P. mirabilis*, a well-recognized cause of urinary tract and wound infections [3,23,24].

A major finding of this study was the high prevalence of ESBL-producing isolates, with 60.6% (60/99) of isolates confirmed phenotypically. Among isolates screened for ESBL production, this corresponded to 63.2% (60/95), indicating widespread dissemination of ESBL-mediated resistance. In addition, 96.0% of isolates were resistant to at least one third- or fourth-generation cephalosporin, highlighting substantial loss of effectiveness of extended-spectrum cephalosporins. These findings are consistent with global trends in ESBL-producing Enterobacterales [1,7] and highlight increasing challenges in empirical therapy. This has important clinical implications, as it limits the effectiveness of commonly used empirical treatment regimens and increases reliance on last-line antibiotics.

Importantly, genomic analysis revealed that ESBL production in this collection was dominated by PER- and VEB-type enzymes, particularly *bla*_PER-13_ and *bla*_VEB-6_. Although CTX-M enzymes are globally predominant, regional emergence of PER- and VEB-type ESBLs has been reported in Africa and other regions [6,7]. The predominance of these non-CTX-M ESBL families in the present study is consistent with possible localized dissemination of resistance determinants within the hospital environment. Similar patterns of ESBL dissemination have been reported in sub-Saharan Africa and other regions [25–28].

The frequent co-occurrence of ESBL genes with narrow-spectrum β-lactamases such as *bla*_TEM-1_ and *bla*_OXA-1_ indicates accumulation of multiple resistance determinants within individual isolates. This layered resistance profile may contribute to broader β-lactam resistance and reduced effectiveness of β-lactam/β-lactamase inhibitor combinations [6,7].

Although partial concordance was observed between phenotypic and genotypic ESBL detection (overall agreement, 68.0%), several discordant isolates were identified. These findings may reflect limitations of phenotypic ESBL detection, differences in gene expression, or resistance mechanisms that were not identified by the current genomic analysis, as previously described for genotype–phenotype discordance in antimicrobial resistance testing [29]. The observed specificity and negative predictive value should be interpreted with caution because whole-genome sequencing was performed on a purposively selected subset enriched for ESBL-producing and/or carbapenemase-producing phenotypes to capture diversity in specimen type, ward/source, multidrug resistance profile, and antimicrobial resistance characteristics, resulting in only three isolates without detectable ESBL-encoding genes and no true-negative isolates. Furthermore, no acquired carbapenemase genes were identified among the phenotypically mCIM-positive isolates, indicating phenotype–genotype discordance for carbapenemase detection. This discrepancy may reflect non-carbapenemase mechanisms of carbapenem resistance, including ESBL or AmpC production combined with reduced membrane permeability or efflux mechanisms, or carbapenemase determinants that were not detected by the current genomic analysis [8,30]. These findings emphasize the importance of integrating phenotypic and genomic approaches [8].

The overall level of multidrug resistant was remarkably high, with 99.0% of isolates meeting the MDR definition [14]. The predominance of 5R and 6R resistance profiles reflects accumulation of resistance across multiple antimicrobial classes, significantly limiting therapeutic options. Similar trends have been reported globally in multidrug-resistant Gram-negative pathogens [1]. The observed co-resistance to non-β-lactam antibiotics further suggests co-selection of resistance determinants, likely mediated by plasmid-associated gene clusters.

MLST and phylogenetic analyses demonstrated moderate genetic heterogeneity, with nine sequence types identified and ST135 as the predominant lineage. These findings are consistent with the possible circulation of multiple resistant lineages. However, because pairwise SNP distance analysis, detailed epidemiological data, and transmission analyses were not performed, direct transmission or endemic circulation cannot be inferred. Comparable patterns of genetic diversity and population structure have been reported in recent genomic studies of Proteus mirabilis [10,31].

The distribution of ESBL genes across sequence types and hospital wards suggests a possible role for mobile genetic elements in shaping resistance patterns. Plasmid replicon analysis identified IncQ1 as the predominant plasmid replicon among the sequenced isolates, consistent with the recognized role of plasmids in the dissemination of antimicrobial resistance genes and the broad host range reported for IncQ1 plasmids [32,33]. IncQ1 and Col-type plasmid replicons were detected; however, plasmid reconstruction, gene–plasmid co-localization analysis, conjugation experiments, and long-read sequencing were not performed. Therefore, the genomic data support only the presence of plasmid replicons and do not permit inference regarding the plasmid location or dissemination of specific antimicrobial resistance genes. Additional studies using long-read sequencing or plasmid reconstruction approaches are required to determine the association between resistance genes and individual plasmids.

The predominance of flagellar and cell surface-associated virulence genes underscores the central role of motility and host interaction in the pathogenicity of *Proteus mirabilis*. Flagellar systems are essential for swarming motility, biofilm formation, and colonization of the urinary tract, which represent key pathogenic traits of this organism [3,34]. The limited detection of advanced virulence determinants, such as type VI secretion system components, suggests that pathogenicity in this setting is primarily driven by conserved, classical mechanisms rather than specialized secretion systems [4]. Importantly, the coexistence of virulence-associated genes with extensive antimicrobial resistance highlights the potential emergence of well-adapted strains capable of persistence, transmission, and therapeutic failure within the hospital environment [1].

In addition to antibiotic resistance determinants, the widespread presence of heavy metal and biocide resistance genes suggests potential co-selection mechanisms within the hospital environment. Such co-selection has been recognized as an important factor in maintaining antimicrobial resistance even in the absence of antibiotic pressure [35].

Overall, the findings indicate that multidrug-resistant *P. mirabilis* in this setting is characterized by clonal diversity and the presence of multiple plasmid replicon types. These findings indicate the presence of multiple multidrug-resistant lineages within the study population; however, additional genomic and epidemiological investigations, including pairwise SNP analyses and epidemiological linkage data, are required to evaluate possible transmission dynamics within the hospital. However, additional genomic analyses, including plasmid reconstruction and gene–plasmid co-localization studies, are required to determine the contribution of specific plasmids to the dissemination of resistance determinants.

## 5 Conclusion

This study demonstrates a high burden of multidrug-resistant *P. mirabilis* in a military referral hospital in Ethiopia, characterized by widespread resistance to extended-spectrum cephalosporins and a high proportion of ESBL-producing isolates. Genomic analysis revealed diverse ESBL determinants dominated by *blaPER-13* and *blaVEB-6*, along with moderate clonal diversity and the presence of multiple plasmid replicon types, particularly IncQ1.

The coexistence of multiple resistance determinants across genetically diverse lineages suggests potential circulation of multidrug-resistant *P. mirabilis* within the hospital environment; however, additional genomic and epidemiological investigations are required to confirm transmission patterns.

These findings highlight the urgent need for strengthened antimicrobial stewardship, enhanced infection prevention strategies, and integration of genomic surveillance into routine hospital microbiology practice to limit further dissemination of multidrug-resistant pathogens in military and comparable healthcare settings. These findings contribute to the limited genomic data on *P. mirabilis* in sub-Saharan Africa and provide a foundation for future surveillance studies.

## 6 Limitations

This study provides important insights into the resistance landscape of *P. mirabilis* in a military referral hospital. However, certain limitations may be considered when interpreting the findings. Whole-genome sequencing was performed on a subset of isolates, which may limit the generalizability of the genomic findings to the entire collection. Furthermore, whole-genome sequencing was performed on a purposively selected subset of isolates enriched for ESBL- and/or carbapenemase-associated phenotypes. Therefore, the genomic findings may not fully represent the diversity and resistance characteristics of the entire isolate collection. In addition, the use of short-read sequencing precluded complete plasmid reconstruction and detailed characterization of the genetic context of resistance determinants. Although mCIM was performed, eCIM and targeted molecular assays were not conducted, limiting definitive classification of carbapenemase types among phenotypically positive isolates. Finally, as this was a single-center study, the findings may not be fully generalizable to other healthcare settings. An additional limitation of this study is that plasmid analyses were based on short-read whole-genome sequencing data. Consequently, plasmid structures and the co-localization of resistance genes with specific plasmid replicons could not be definitively determined. Long-read sequencing and plasmid reconstruction approaches would be required to confirm plasmid-mediated transmission and to better characterize the genetic context of antimicrobial resistance determinants.

## Supporting information

S1 Table Comparison of the sequenced subset with the complete Proteus mirabilis isolate collection by specimen type, ward/source, ESBL status, carbapenemase status, multidrug resistance profile, and key phenotypic resistance patterns. (DOCX)

S2 Table Gene presence/absence matrix of β-lactamase genes identified among sequenced Proteus mirabilis isolates. (DOCX)

S3 Table Genome assembly quality statistics for sequenced Proteus mirabilis isolates, including genome size, GC content, contig number, N50 values, and BUSCO completeness. (DOCX)
