# Supplementary material for: Genomic epidemiology of multidrug-resistant and ESBL-producing Proteus mirabilis in a military referral hospital in Ethiopia
Source: PLoS One. 2026 Aug 11;21(8):e0355508. doi: 10.1371/journal.pone.0355508 (PMC13460578; doi:10.1371/journal.pone.0355508)
Supplement: Supplementary file 1 [file pone.0355508.s001.docx]

# Supplementary Table S1

Comparison of the characteristics of the complete *Proteus mirabilis* isolate collection (n = 99) and the purposively selected whole-genome sequenced subset (n = 25).

| **Characteristic** | **Full collection (n=99)** | **WGS subset (n=25)** |
| --- | --- | --- |
| **Specimen type** |  |  |
| Urine | 52 (52.5%) | 12 (48.0%) |
| Wound | 42 (42.4%) | 11 (44.0%) |
| Blood | 5 (5.1%) | 2 (8.0%) |
| **Ward/source** |  |  |
| OPD | 30 (30.3%) | 5 (20.0%) |
| Medical (A, B, D, E) | 21 (21.2%) | 6 (24.0%) |
| Surgical (A, B, C, D, E, F, G) | 38 (38.4%) | 10 (40.0%) |
| Orthopedics (A, B, C) | 7 (7.1%) | 3 (12.0%) |
| ICU | 3 (3.0%) | 1 (4.0%) |
| **ESBL phenotype** |  |  |
| ESBL-positive | 60 (60.6%) | 20 (80.0%) |
| ESBL-negative | 39 (39.4%) | 5 (20.0%) |
| **Carbapenemase phenotype (mCIM)** |  |  |
| Positive | 6 (6.1%) | 6 (24.0%) |
| Negative | 93 (93.9%) | 19 (76.0%) |
| **Multidrug resistance (MDR)** |  |  |
| **MDR** | 98 (99.0%) | 25 (100.0%) |
| Non-MDR | 1 (1.0%) | 0 (0.0%) |
| **Key phenotypic resistance patterns‡** |  |  |
| Ceftriaxone (CRO) | 77 (77.8%) | 25 (100.0%) |
| Cefotaxime (CTX) | 83 (83.8%) | 23 (92.0%) |
| Ceftazidime (CAZ) | 75 (75.8%) | 21 (84.0%) |
| Cefepime (FEP) | 67 (67.7%) | 22 (88.0%) |
| Aztreonam (ATM) | 76 (76.8%) | 21 (84.0%) |
| Ciprofloxacin (CIP) | 78 (78.8%) | 19 (76.0%) |
| Gentamicin (CN) | 44 (44.4%) | 20 (80.0%) |
| Meropenem (MRO) | 21 (21.2%) | 9 (36.0%) |

**Abbreviations:** WGS, whole-genome sequencing; ESBL, extended-spectrum β-lactamase; mCIM, modified carbapenem inactivation method; MDR, multidrug-resistant; OPD, outpatient department; ICU, intensive care unit.

‡ Non-susceptibility was defined as resistant plus intermediate (R + I) according to the study's antimicrobial susceptibility interpretation.
