# Supplementary material for: Genomic epidemiology of multidrug-resistant and ESBL-producing Proteus mirabilis in a military referral hospital in Ethiopia
Source: PLoS One. 2026 Aug 11;21(8):e0355508. doi: 10.1371/journal.pone.0355508 (PMC13460578; doi:10.1371/journal.pone.0355508)
Supplement: Supplementary file 2 [file pone.0355508.s002.docx]

**Supplementary Table S2.** Comprehensive Distribution of Non-β-Lactam Resistance, Metal, and Biocide Resistance Genes Identified in Sequenced Proteus mirabilis Isolates (n = 25)

| **Resistance Group** | **ARGs** | **No. of Isolates** | **Percentage (%)** |
| --- | --- | --- | --- |
| Fluoroquinolones (PMQR) | *qnrD1* | 7 | 28.0 |
|  | *aac(6′)-Ib-cr5* | 4 | 16.0 |
|  | *qnrD2* | 1 | 4.0 |
|  | *qnrA1* | 1 | 4.0 |
|  | *qnrD3* | 1 | 4.0 |
| Aminoglycosides | *aadA1* | 20 | 80.0 |
|  | *aph(6)-Id* | 12 | 48.0 |
|  | *aph(3″)-Ib* | 12 | 48.0 |
|  | *aadA5* | 11 | 44.0 |
|  | *ant(2″)-Ia* | 11 | 44.0 |
| Macrolides/Lincosamides | *mph(E)* | 10 | 40.0 |
|  | *lnu(F)* | 10 | 40.0 |
|  | *ere(A)* | 8 | 32.0 |
| Tetracyclines | *tet(J)* | 25 | 100.0 |
|  | *tet(A)* | 6 | 24.0 |
| Sulfonamides | *sul1* | 21 | 84.0 |
|  | *sul2* | 12 | 48.0 |
| Trimethoprim | *dfrA1* | 18 | 72.0 |
|  | *dfrA17* | 11 | 44.0 |
|  | *dfrA5* | 4 | 16.0 |
|  | *dfrA15* | 1 | 4.0 |
| Phenicols | *catA* | 24 | 96.0 |
|  | *catA1* | 16 | 64.0 |
|  | *catB3* | 5 | 20.0 |
| Metal Resistance | *terD* | 25 | 100.0 |
|  | *terZ* | 25 | 100.0 |
|  | *mer operon* | 11 | 44.0 |
| Biocide Resistance | *qacEΔ1* | 15 | 60.0 |
|  | *qacE* | 8 | 32.0 |
