# Supplementary material for: Genomic epidemiology of multidrug-resistant and ESBL-producing Proteus mirabilis in a military referral hospital in Ethiopia
Source: PLoS One. 2026 Aug 11;21(8):e0355508. doi: 10.1371/journal.pone.0355508 (PMC13460578; doi:10.1371/journal.pone.0355508)
Supplement: Supplementary file 3 [file pone.0355508.s003.docx]

# Supplementary Table S3. Genome Assembly and Sequencing Quality Metrics for the 25 Whole-Genome Sequenced Proteus mirabilis Isolates

| **Isolate ID** | **Assembly Size (bp)** | **BUSCO Completeness (%)** | **N50 (bp)** | **No. of Contigs** | **GC Content (%)** | **R1 Mean Quality (Phred)** | **R1 Mean Read Length (bp)** | **R2 Mean Quality (Phred)** | **R2 Mean Read Length (bp)** |
| --- | --- | --- | --- | --- | --- | --- | --- | --- | --- |
| EPHIPM001 | 4,115,748 | 99.6 | 185,839 | 48 | 38.69 | 30.35 | 136.97 | 29.87 | 136.73 |
| EPHIPM002 | 4,018,623 | 99.6 | 194,217 | 49 | 38.85 | 30.54 | 135.64 | 29.83 | 136.32 |
| EPHIPM003 | 4,081,307 | 99.6 | 155,733 | 69 | 38.75 | 30.40 | 136.53 | 29.98 | 136.61 |
| EPHIPM004 | 4,063,410 | 99.6 | 193,360 | 65 | 39.11 | 30.37 | 136.77 | 29.72 | 136.92 |
| EPHIPM005 | 4,103,000 | 99.6 | 158,241 | 61 | 38.79 | 30.11 | 137.84 | 29.60 | 137.62 |
| EPHIPM006 | 4,255,900 | 98.6 | 111,403 | 159 | 39.28 | 30.40 | 136.71 | 29.78 | 136.91 |
| EPHIPM007 | 3,953,807 | 99.6 | 173,862 | 56 | 38.78 | 30.32 | 137.92 | 29.75 | 138.00 |
| EPHIPM008 | 3,807,902 | 99.6 | 186,511 | 45 | 38.75 | 30.51 | 137.48 | 29.96 | 137.66 |
| EPHIPM009 | 4,007,385 | 99.5 | 174,896 | 84 | 39.03 | 30.51 | 135.88 | 29.91 | 136.45 |
| EPHIPM010 | 4,162,836 | 99.6 | 207,066 | 60 | 38.80 | 29.92 | 135.61 | 30.17 | 135.05 |
| EPHIPM011 | 4,026,240 | 99.6 | 201,205 | 67 | 39.12 | 30.42 | 137.48 | 29.90 | 137.51 |
| EPHIPM012 | 4,065,553 | 99.6 | 168,049 | 58 | 38.84 | 30.39 | 135.51 | 29.97 | 135.49 |
| EPHIPM013 | 3,972,860 | 99.6 | 218,681 | 66 | 38.78 | 30.54 | 136.05 | 30.08 | 136.23 |
| EPHIPM014 | 4,096,183 | 99.6 | 185,215 | 79 | 39.04 | 30.24 | 139.48 | 29.40 | 140.00 |
| EPHIPM015 | 4,168,649 | 99.6 | 194,455 | 60 | 38.84 | 30.44 | 137.06 | 29.92 | 137.41 |
| EPHIPM016 | 4,088,927 | 99.6 | 138,332 | 102 | 39.12 | 30.45 | 135.38 | 29.84 | 136.03 |
| EPHIPM017 | 4,033,428 | 99.6 | 189,761 | 99 | 39.24 | 31.53 | 118.97 | 29.92 | 119.83 |
| EPHIPM018 | 4,078,178 | 99.6 | 164,639 | 65 | 38.75 | 31.30 | 122.24 | 29.52 | 123.07 |
| EPHIPM019 | 4,669,931 | 99.6 | 107,403 | 424 | 39.31 | 31.14 | 121.47 | 29.14 | 123.04 |
| EPHIPM020 | 4,078,092 | 99.6 | 158,339 | 70 | 38.75 | 31.21 | 121.68 | 29.48 | 123.08 |
| EPHIPM021 | 3,917,598 | 99.5 | 196,004 | 67 | 38.99 | 31.16 | 115.86 | 29.42 | 117.90 |
| EPHIPM022 | 4,058,749 | 99.6 | 188,529 | 66 | 39.10 | 31.07 | 121.26 | 29.25 | 123.24 |
| EPHIPM023 | 4,074,579 | 99.6 | 169,025 | 69 | 38.74 | 31.42 | 118.43 | 29.66 | 119.93 |
| EPHIPM024 | 4,073,621 | 99.6 | 150,516 | 72 | 38.82 | 31.12 | 121.02 | 29.42 | 121.98 |
| EPHIPM025 | 4,186,732 | 99.6 | 112,953 | 108 | 38.78 | 31.03 | 121.64 | 29.21 | 123.59 |

**Footnote**: BUSCO, Benchmarking Universal Single-Copy Orthologs; N50, contig length at which 50% of the assembled genome is contained in contigs of equal or greater length; GC, guanine-cytosine content; R1 and R2, forward and reverse Illumina reads, respectively. Mean quality scores represent Phred quality scores, and mean read lengths represent the average read length reported by the sequencing quality assessment.
