# Supplementary material for: Genomic epidemiology of multidrug-resistant and ESBL-producing Proteus mirabilis in a military referral hospital in Ethiopia
Source: PLoS One. 2026 Aug 11;21(8):e0355508. doi: 10.1371/journal.pone.0355508 (PMC13460578; doi:10.1371/journal.pone.0355508)
Supplement: Supplementary file 4 [file pone.0355508.s005.docx]

**Reviewer #2 – General Comment**

The novelty of this study lies in its integrated genomic and phenotypic characterization of multidrug-resistant Proteus mirabilis isolates obtained from a military hospital environment, with particular emphasis on the diversity of ESBL determinants, plasmid-mediated dissemination, and population structure. Although antimicrobial resistance in P. mirabilis has been widely reported, comprehensive whole-genome epidemiological analyses from military healthcare settings remain limited.

The bioinformatic component appears to be the strongest aspect of the study; however, in my opinion, the overall novelty of the research is currently insufficient for publication in its present form. The study focuses primarily on a single area of investigation, which limits the broader scientific impact of the findings. Overall, the manuscript presents data mainly in an epidemiological context and offers limited scientific novelty.

**Response:**

We thank the reviewer for this thoughtful assessment. We acknowledge that the study is primarily epidemiological in nature and does not aim to investigate molecular mechanisms through experimental approaches. However, we respectfully believe that the study provides important novel data from a setting where genomic surveillance information remains extremely limited.

To strengthen the contribution of the manuscript, we have revised the Introduction, Discussion, and Conclusion sections to better highlight the study’s novelty and significance. Specifically, the study integrates phenotypic resistance characterization with whole-genome sequencing, plasmid replicon analysis, virulence profiling, multilocus sequence typing, and phylogenetic analysis of multidrug-resistant P. mirabilis isolates recovered from a military referral hospital in Ethiopia. To our knowledge, this represents one of the first comprehensive genomic investigations of multidrug-resistant P. mirabilis from a military healthcare setting in Ethiopia and provides baseline genomic surveillance data from a region where such information is scarce.

We have also revised the Discussion to place the findings within the broader context of antimicrobial resistance surveillance, infection prevention, and genomic epidemiology in low-resource settings.

**Changes made:**

• The Introduction was revised to better emphasize the knowledge gap addressed by the study.

• The Discussion was expanded to highlight the significance of integrating phenotypic and genomic data from a military healthcare setting.

• The Conclusion was revised to better articulate the study’s contribution to genomic surveillance and antimicrobial resistance monitoring in Ethiopia and sub-Saharan Africa.
