# Supplementary material for: Genomic epidemiology of multidrug-resistant and ESBL-producing Proteus mirabilis in a military referral hospital in Ethiopia
Source: PLoS One. 2026 Aug 11;21(8):e0355508. doi: 10.1371/journal.pone.0355508 (PMC13460578; doi:10.1371/journal.pone.0355508)
Supplement: Supplementary file 5 [file pone.0355508.s006.docx]

**Reviewer Comment:**

First, the manuscript still does not provide a clear comparison between the 25 sequenced isolates and the full 99-isolate collection. Because the WGS subset was purposively selected and enriched for ESBL- and/or carbapenemase-associated phenotypes, the authors should add a supplementary table comparing the sequenced subset with the full collection by specimen type, ward/source, ESBL status, carbapenemase status, MDR profile, and key phenotypic resistance patterns.

**Response:**

We thank the reviewer for this valuable suggestion. We have addressed this comment by adding a new **Supplementary Table S1**, which provides a direct comparison between the complete Proteus mirabilis isolate collection (n = 99) and the purposively selected whole-genome sequenced subset (n = 25). The table summarizes specimen type, ward/source, ESBL phenotype, carbapenemase phenotype, multidrug-resistant (MDR) status, and key phenotypic antimicrobial resistance patterns. In addition, the Methods section (Section 2.3, Clinical Specimens and Isolate Selection) has been revised to clarify the rationale for selecting the sequenced isolates and to direct readers to Supplementary Table S1.

**Reviewer Comment:**

Second, the sequencing-quality information remains incomplete. The added assembly metrics are useful, but Supplementary Table S2 should also include total reads, coverage depth, isolate-level SRA run IDs or assembly accessions, and species confirmation/contamination assessment if available. These details are important because the manuscript discusses genotype–phenotype discordance, and readers need enough information to assess whether gene absence may be biological or technical.

**Response:**

**We sincerely thank the reviewer for this constructive suggestion. We have revised Supplementary Table S3 to provide more comprehensive isolate-level sequencing and assembly quality information. In addition to the assembly quality metrics (assembly size, BUSCO completeness, N50, number of contigs, and GC content), the revised table now includes sequencing quality metrics, specifically the mean R1 and R2 Phred quality scores and mean R1 and R2 read lengths for each sequenced isolate.**

**Regarding the additional information requested, total read counts, sequencing coverage depth, isolate-level SRA run IDs or assembly accessions, and formal species confirmation or contamination assessment were not generated or recorded as part of the original sequencing and bioinformatics workflow and therefore are not available for retrospective inclusion. Species identification was confirmed prior to sequencing using MALDI-TOF MS, and the high assembly completeness, expected genome sizes, GC content, and BUSCO completeness values support the quality and identity of the assembled genomes. We have, however, provided all available isolate-level sequencing and assembly quality metrics to improve transparency and to facilitate assessment of the quality and reliability of the genomic data used in this study.**

**Reviewer Comment**

Third, the ESBL genotype–phenotype concordance analysis has not been adequately addressed. The previous review requested a 2 × 2 table with sensitivity, specificity, positive predictive value, negative predictive value, and overall agreement. The revised manuscript provides a narrative description but does not provide this table. Based on the numbers reported, 20 sequenced isolates were phenotypically ESBL-positive, 17 of these carried ESBL-associated genes, and five phenotypically ESBL-negative isolates carried ESBL-associated genes. Therefore, the overall agreement appears to be 17/25, or 68%, and should not be described as high or substantial without qualification. This analysis should be clearly presented and interpreted cautiously.

**Response:**

We sincerely thank the reviewer for this valuable suggestion. In response, we revised the phenotypic–genotypic concordance analysis in Section 3.6. A new Table 4 has been added presenting the requested 2 × 2 contingency table comparing phenotypic ESBL detection by the modified double-disk synergy test (MDDST) with ESBL-encoding genes identified by whole-genome sequencing. In addition, we calculated and reported the diagnostic performance of phenotypic ESBL detection, including sensitivity, specificity, positive predictive value (PPV), negative predictive value (NPV), and overall agreement. Consistent with the reviewer's observation, the overall agreement was 68.0% (17/25). Accordingly, we revised the interpretation throughout the manuscript by replacing the previous description of "substantial agreement" with "partial concordance" and expanded the Discussion to acknowledge the observed discordance between phenotypic and genotypic findings. We also clarified that the observed specificity and negative predictive value should be interpreted with caution because whole-genome sequencing was performed on a purposively selected subset enriched for ESBL-producing and/or carbapenemase-producing phenotypes, resulting in a limited number of isolates without detectable ESBL-encoding genes and no true-negative isolates. Furthermore, we discussed potential explanations for the observed discordance, including limitations of phenotypic detection, differences in gene expression, and resistance mechanisms that may not have been identified by the current genomic analysis. To better support this interpretation, we replaced the previous citation with a more appropriate and up-to-date reference addressing genotype–phenotype discordance in antimicrobial resistance testing.

**Reviewer Comment:**

Fourth, carbapenemase genotype–phenotype interpretation remains underdeveloped. The authors clarified that mCIM was performed and that eCIM was not used, which is helpful. However, the manuscript should clearly state whether carbapenemase genes were detected among the six mCIM-positive isolates. If no carbapenemase genes were detected, this should be explicitly discussed as phenotype–genotype discordance. If genes were detected, they should be reported isolate by isolate.

**Response:**

We sincerely thank the reviewer for this valuable comment. We have revised the manuscript to clarify the relationship between phenotypic carbapenemase detection and whole-genome sequencing results. We now explicitly state in the Results section that **no acquired carbapenemase genes were identified among the sequenced isolates, including those that were phenotypically positive by the modified carbapenem inactivation method (mCIM).** This finding has been incorporated into the β-lactamase and ESBL gene section (3.8).

In addition, we have expanded the Discussion to acknowledge this **phenotype–genotype discordance** and to discuss potential explanations, including non-carbapenemase mechanisms of carbapenem resistance (e.g., ESBL or AmpC production combined with reduced membrane permeability or efflux mechanisms), as well as the possibility that carbapenemase determinants were not detected by the current genomic analysis. These revisions provide a clearer interpretation of the phenotypic and genomic findings and directly address the reviewer's concern.

**Reviewer Comment:**

Fifth, the antimicrobial susceptibility testing methods still require more complete reproducibility details. The Methods should list the full antibiotic panel, disk potencies, interpretive criteria, and whether any antibiotics were excluded or interpreted cautiously because of intrinsic resistance or species-specific considerations for *Proteus mirabilis*.

**Response:**

We sincerely thank the reviewer for this helpful suggestion. We have revised the Antimicrobial Susceptibility Testing section of the Methods (2.5) to improve the reproducibility of the study. The revised text now includes the complete panel of antimicrobial agents tested, together with their corresponding disk potencies. We have also clarified that antimicrobial susceptibility results were interpreted according to the Clinical and Laboratory Standards Institute (CLSI) M100, 33rd edition guidelines and that isolates categorized as intermediate or resistant were considered non-susceptible for comparative analyses. In addition, we retained the definition of multidrug resistance (MDR) according to the international consensus criteria proposed by Magiorakos et al. These revisions provide the methodological detail requested by the reviewer and improve the transparency and reproducibility of the antimicrobial susceptibility testing procedures**.**

**Reviewer Comment:**

Sixth, some interpretation of phylogeny and hospital circulation remains too strong. Without pairwise SNP distances, sampling dates, patient movement data, ward overlap, or defined genomic clusters, the authors should avoid wording that suggests proven endemic circulation or transmission. Wording such as “is consistent with possible circulation” or “suggests possible lineage expansion” would be more appropriate**.**

**Response:**

We thank the reviewer for this valuable comment. We agree that our study was not designed to infer transmission dynamics and did not include pairwise SNP distance analysis, detailed sampling dates, patient movement information, ward overlap analysis, or predefined genomic transmission clusters. Accordingly, we have revised the Discussion to adopt more cautious language when interpreting the phylogenetic findings. Statements implying clonal spread or hospital circulation have been replaced with more conservative wording such as **"suggests possible lineage expansion," "is consistent with possible localized dissemination,"** and **"is consistent with the possible circulation of multiple resistant lineages."** We also explicitly state that additional genomic and epidemiological investigations are required to confirm transmission dynamics. These revisions ensure that our conclusions remain fully supported by the available data.

**Reviewer Comment:**

Seventh, plasmid interpretation should remain limited to replicon detection. The authors correctly acknowledge that short-read data cannot confirm plasmid structure or gene–plasmid co-localization. Any remaining wording implying plasmid-mediated dissemination of specific ESBL genes should be removed unless supported by contig-level co-localization, plasmid reconstruction, MOB-suite analysis, long-read sequencing, or conjugation data.

**Response:**
We thank the reviewer for this important comment. We agree that short-read whole-genome sequencing and plasmid replicon detection alone cannot establish plasmid structure or confirm the co-localization of antimicrobial resistance genes on specific plasmids. Accordingly, we have revised the Discussion to restrict our interpretation to the detection of plasmid replicons and removed wording that could imply plasmid-mediated dissemination of specific ESBL genes. We now explicitly state that plasmid reconstruction, gene–plasmid co-localization analyses, conjugation experiments, and long-read sequencing were not performed, and that additional studies are required to determine the relationship between resistance genes and individual plasmids.

**Reviewer Comment:**

Eighth, the statistical analysis remains limited. The authors now state that Fisher’s exact test was used and that comparisons involving carbapenemase-positive isolates are exploratory, which is appropriate. However, p-values from very small groups should not be overemphasized. Where feasible, odds ratios with confidence intervals should be added for the main comparisons, or the authors should explicitly state that effect-size estimates were not reported because of instability due to sparse cells.

**Response:**

We sincerely thank the reviewer for this valuable suggestion. We agree that effect-size measures such as **odds ratios (ORs) with 95% confidence intervals** can provide additional information beyond p-values when sample sizes are adequate. However, because several contingency tables in our study, particularly those involving the **six carbapenemase-positive isolates**, contained sparse cells and small group sizes, the resulting ORs and confidence intervals would be unstable and potentially misleading. Therefore, we did not calculate or report these effect-size estimates. Instead, we have revised the **Statistical Analysis** section to explicitly state that **odds ratios and corresponding 95% confidence intervals were not reported because of instability resulting from sparse cells**, and that comparisons involving carbapenemase-positive isolates should be interpreted as exploratory. We believe this approach provides a more appropriate and transparent interpretation of the statistical analyses while avoiding overinterpretation of results derived from very small sample sizes.

**Reviewer Comment:**

Ninth, the figures remain a concern. Although the authors state that the figures were improved, the submitted versions still appear cropped or difficult to read, especially the phylogeny and virulence heatmap. Figure 2 should be regenerated with readable isolate labels, clear metadata tracks, larger legends, and a caption explaining all colour annotations. Figure 3 should be presented as a clean heatmap with legible gene names and isolate labels. Final figure files should be checked at publication size.

**Response:**

We sincerely thank the reviewer for this valuable suggestion. In response, we have regenerated and carefully revised Figures 2 and 3 to improve their readability and presentation quality.

For Figure 2, the phylogenetic tree has been reformatted by increasing the font size of the isolate labels, metadata track labels, and legend, improving the overall layout, and ensuring that the figure remains clear and legible at publication size. The figure caption has also been expanded to clearly explain all metadata tracks and colour annotations.

For Figure 3, the virulence gene heatmap has been regenerated with improved formatting, including larger isolate labels, gene names, and legend text, while preserving the original data and layout. The "No. Found" values were recalculated and verified directly from the gene presence/absence matrix to ensure complete consistency with the underlying data. The spacing and overall layout were also optimized to improve readability at publication size.

Finally, all figure files have been exported as high-resolution TIFF images (600 dpi) and carefully checked at their intended publication size to ensure that all labels, legends, and graphical elements are clear and fully legible.

**Reviewer Comment 10:**

The references and formatting still need careful checking. Some citations appear mismatched to the statements they support. For example, a statement on Proteus mirabilis flagellar systems and virulence appears to cite a reference concerning *E. coli* CTX-M-27 plasmids, which does not support that claim. The authors should re-audit all references, especially those cited for Proteus mirabilis virulence, plasmids, ESBL epidemiology, and recent 2025/2026 claims.

**Response:**
Response:

We sincerely thank the reviewer for this careful assessment. In response, we comprehensively re-audited all in-text citations and the reference list to ensure that each reference directly supports the corresponding statement. Specifically, the citation associated with the discussion of Proteus mirabilis flagellar systems, swarming motility, biofilm formation, and urinary tract colonization was corrected by replacing the unrelated reference concerning Escherichia coli CTX-M-27 plasmids with appropriate Proteus mirabilis-specific references. We also revised the wording of several Discussion paragraphs to better align the interpretation with the supporting literature. Specifically, the discussions of phylogenetic diversity, ESBL epidemiology, plasmid biology, and carbapenem resistance mechanisms were updated, and the corresponding citations were replaced with more appropriate and recent references, including current genomic epidemiology studies of P. mirabilis. Furthermore, all recent references (2025–2026) and bibliographic details were carefully verified to ensure that every citation accurately supports the associated statement and that the reference list is fully consistent throughout the manuscript.

**Reviewer Comment:**

Finally, the manuscript requires a careful final language and formatting edit. There are still missing spaces, inconsistent section numbering, and awkward phrases such as “extensive multidrug-resistant associated.” The plasmid section numbering also appears inconsistent. These issues should be corrected before acceptance.

**Response:**

We sincerely thank the reviewer for this careful observation. In response, we performed a comprehensive language and formatting review of the entire manuscript. Grammar, spelling, punctuation, and readability were carefully revised throughout the text. Missing spaces, typographical errors, and inconsistent formatting were corrected. Section numbering, including the plasmid-related section, was reviewed and corrected to ensure consistency. Awkward or unclear expressions, including the phrase **“extensive multidrug-resistant associated,”** were revised for clarity and scientific accuracy. A final editorial review was performed to ensure consistency in language, formatting, terminology, and overall presentation throughout the manuscript.
