# Supplementary material for: Genomic epidemiology of multidrug-resistant and ESBL-producing Proteus mirabilis in a military referral hospital in Ethiopia
Source: PLoS One. 2026 Aug 11;21(8):e0355508. doi: 10.1371/journal.pone.0355508 (PMC13460578; doi:10.1371/journal.pone.0355508)
Supplement: Peer review history

# Peer review: Decision Letter 0

**DOI:** 10.1371/journal.pone.0355508.r001

**Authors:** Zhi Ruan

27 May 2026

*Proteus mirabilis*

Dear Dr. Yusuf,

Thank you for submitting your manuscript to PLOS ONE. After careful consideration, we feel that it has merit but does not fully meet PLOS ONE’s publication criteria as it currently stands. Therefore, we invite you to submit a revised version of the manuscript that addresses the points raised during the review process.

Please submit your revised manuscript by Jul 11 2026 11:59PM. If you will need more time than this to complete your revisions, please reply to this message or contact the journal office at plosone@plos.org. When you're ready to submit your revision, log on to https://www.editorialmanager.com/pone/ and select the 'Submissions Needing Revision' folder to locate your manuscript file.

- A letter that responds to each point raised by the academic editor and reviewer(s). You should upload this letter as a separate file labeled 'Response to Reviewers'.
- A marked-up copy of your manuscript that highlights changes made to the original version. You should upload this as a separate file labeled 'Revised Manuscript with Track Changes'.
- An unmarked version of your revised paper without tracked changes. You should upload this as a separate file labeled 'Manuscript'.

If you would like to make changes to your financial disclosure, please include your updated statement in your cover letter. Guidelines for resubmitting your figure files are available below the reviewer comments at the end of this letter.

If applicable, we recommend that you deposit your laboratory protocols in protocols.io to enhance the reproducibility of your results. Protocols.io assigns your protocol its own identifier (DOI) so that it can be cited independently in the future. For instructions see: https://journals.plos.org/plosone/s/submission-guidelines#loc-laboratory-protocols. Additionally, PLOS ONE offers an option for publishing peer-reviewed Lab Protocol articles, which describe protocols hosted on protocols.io. Read more information on sharing protocols at https://plos.org/protocols?utm_medium=editorial-email&utm_source=authorletters&utm_campaign=protocols.

As the corresponding author, your ORCID iD is verified in the submission system and will appear in the published article. PLOS supports the use of ORCID, and we encourage all coauthors to register for an ORCID iD and use it as well. Please encourage your coauthors to verify their ORCID iD within the submission system before final acceptance, as unverified ORCID iDs will not appear in the published article. *Only*  the individual author can complete the verification step; PLOS staff *cannot*  verify ORCID iDs on behalf of authors.

We look forward to receiving your revised manuscript.

Kind regards,

Zhi Ruan, Ph.D.

Academic Editor

PLOS One

Journal Requirements:

When submitting your revision, we need you to address these additional requirements.

1. Please ensure that your manuscript meets PLOS ONE's style requirements, including those for file naming. The PLOS ONE style templates can be found at https://journals.plos.org/plosone/s/file?id=wjVg/PLOSOne_formatting_sample_main_body.pdf and https://journals.plos.org/plosone/s/file?id=ba62/PLOSOne_formatting_sample_title_authors_affiliations.pdf

2. Thank you for stating the following in the Competing Interests section:

[The authors have declared that no competing interests exist.].

We note that one or more of the authors are employed by a commercial company: Wudassie Diagnostic Centre.

1. Please provide an amended Funding Statement declaring this commercial affiliation, as well as a statement regarding the Role of Funders in your study. If the funding organization did not play a role in the study design, data collection and analysis, decision to publish, or preparation of the manuscript and only provided financial support in the form of authors' salaries and/or research materials, please review your statements relating to the author contributions, and ensure you have specifically and accurately indicated the role(s) that these authors had in your study. You can update author roles in the Author Contributions section of the online submission form.

Please also include the following statement within your amended Funding Statement.

“The funder provided support in the form of salaries for authors [insert relevant initials], but did not have any additional role in the study design, data collection and analysis, decision to publish, or preparation of the manuscript. The specific roles of these authors are articulated in the ‘author contributions’ section.”

If your commercial affiliation did play a role in your study, please state and explain this role within your updated Funding Statement.

2. Please also provide an updated Competing Interests Statement declaring this commercial affiliation along with any other relevant declarations relating to employment, consultancy, patents, products in development, or marketed products, etc.

Within your Competing Interests Statement, please confirm that this commercial affiliation does not alter your adherence to all PLOS ONE policies on sharing data and materials by including the following statement: "This does not alter our adherence to PLOS ONE policies on sharing data and materials.” (as detailed online in our guide for authors http://journals.plos.org/plosone/s/competing-interests) . If this adherence statement is not accurate and there are restrictions on sharing of data and/or materials, please state these. Please note that we cannot proceed with consideration of your article until this information has been declared.

Please include both an updated Funding Statement and Competing Interests Statement in your cover letter. We will change the online submission form on your behalf.

3. Please amend either the title on the online submission form (via Edit Submission) or the title in the manuscript so that they are identical.

4. Please amend either the abstract on the online submission form (via Edit Submission) or the abstract in the manuscript so that they are identical.

5. Your ethics statement should only appear in the Methods section of your manuscript. If your ethics statement is written in any section besides the Methods, please move it to the Methods section and delete it from any other section. Please ensure that your ethics statement is included in your manuscript, as the ethics statement entered into the online submission form will not be published alongside your manuscript.

6. Please ensure that you refer to Figure 2 in your text as, if accepted, production will need this reference to link the reader to the figure.

7. Please remove your figures from within your manuscript file, leaving only the individual TIFF/EPS image files, uploaded separately. These will be automatically included in the reviewers’ PDF.

8. We note that there is identifying data in Table 1. Prior to sharing human research participant data, authors should consult with an ethics committee to ensure data are shared in accordance with participant consent and all applicable local laws.

Data sharing should never compromise participant privacy. It is therefore not appropriate to publicly share personally identifiable data on human research participants. The following are examples of data that should not be shared:

-Name, initials, physical address

-Ages more specific than whole numbers

-Internet protocol (IP) address

-Specific dates (birth dates, death dates, examination dates, etc.)

-Contact information such as phone number or email address

-Location data

-ID numbers that seem specific (long numbers, include initials, titled “Hospital ID”) rather than random (small numbers in numerical order)

Data that are not directly identifying may also be inappropriate to share, as in combination they can become identifying. For example, data collected from a small group of participants, vulnerable populations, or private groups should not be shared if they involve indirect identifiers (such as sex, ethnicity, location, etc.) that may risk the identification of study participants.

Additional guidance on preparing raw data for publication can be found in our Data Policy (https://journals.plos.org/plosone/s/data-availability#loc-human-research-participant-data-and-other-sensitive-data) and in the following article: http://www.bmj.com/content/340/bmj.c181.long.

Please remove or anonymize all personal information (location in caption, population type), ensure that the data shared are in accordance with participant consent, and add an anonymized version to your manuscript file.

9. We note you have included a table to which you do not refer in the text of your manuscript. Please ensure that you refer to Table 4 in your text; if accepted, production will need this reference to link the reader to the Table.

10. Please include captions for your Supporting Information files at the end of your manuscript, and update any in-text citations to match accordingly. Please see our Supporting Information guidelines for more information: http://journals.plos.org/plosone/s/supporting-information.

If the reviewer comments include a recommendation to cite specific previously published works, please review and evaluate these publications to determine whether they are relevant and should be cited. There is no requirement to cite these works unless the editor has indicated otherwise.

[Note: HTML markup is below. Please do not edit.]

Reviewers' comments:

Reviewer's Responses to Questions

**Comments to the Author**

1. Is the manuscript technically sound, and do the data support the conclusions?

Reviewer #1: Yes

Reviewer #2: Yes

**********

2. Has the statistical analysis been performed appropriately and rigorously?

Reviewer #1: Yes

Reviewer #2: Yes

**********

3. Have the authors made all data underlying the findings in their manuscript fully available?

The PLOS Data policy

Reviewer #1: Yes

Reviewer #2: No

**********

4. Is the manuscript presented in an intelligible fashion and written in standard English?

Reviewer #1: Yes

Reviewer #2: Yes

**********

Reviewer #1: Overall assessment

This manuscript addresses an important and underrepresented topic: the phenotypic and genomic epidemiology of multidrug-resistant Proteus mirabilis in a military referral hospital in Ethiopia. The study is timely and potentially valuable because genomic data on P. mirabilis from sub-Saharan Africa remain limited, and the authors combine antimicrobial susceptibility testing, ESBL/carbapenemase phenotyping, whole-genome sequencing, MLST, resistance gene detection, plasmid replicon analysis, virulence profiling, and phylogenomics. The manuscript reports a very high MDR burden among 99 clinical isolates, with 25 selected isolates undergoing WGS, and identifies ESBL determinants including blaPER-13, blaVEB-6, and blaCTX-M-65, as well as multiple non-β-lactam resistance genes and plasmid replicons .

However, several substantial issues need to be addressed before the manuscript can be considered suitable for publication in PLOS ONE. The main concerns relate to the representativeness of the sequenced subset, insufficient methodological detail, overinterpretation of genomic epidemiology and plasmid-mediated transmission, incomplete reporting of sequencing quality metrics, unclear genotype–phenotype concordance analysis, limited statistical treatment, and figure quality. The study has merit, but the current version requires major revision to strengthen reproducibility, interpretation, and presentation.

Major comments

1. The study includes 99 non-duplicate P. mirabilis isolates, but only 25 were sequenced. The authors state that these were “representative ESBL- and/or carbapenemase-producing isolates” chosen to represent specimen types and hospital wards within sequencing capacity. This selection is understandable, but it introduces enrichment bias because the sequenced subset is not a random or complete representation of the full isolate collection.

The manuscript should clearly present how the 25 isolates compare with the full 99-isolate collection in terms of specimen type, ward, ESBL status, carbapenemase status, resistance profile, age group, and patient category. A table comparing the sequenced and non-sequenced isolates would greatly improve transparency.

The authors should also revise language suggesting that genomic findings reflect the entire hospital population. Statements such as “endemic hospital circulation,” “plasmid-mediated dissemination,” and “horizontal dissemination across hospital units” should be presented more cautiously unless supported by denser sampling, temporal clustering, SNP distance thresholds, plasmid reconstruction, or epidemiological linkage data.

2. The methods mention FastQC, fastp, Shovill/SKESA, QUAST, BUSCO, AMRFinderPlus, MLST, Snippy, IQ-TREE, and ABRicate. However, the manuscript does not provide isolate-level sequencing quality metrics. For reproducibility and confidence in downstream analyses, the authors should include a supplementary table with, at minimum:

coverage depth, total reads, genome size, number of contigs, N50, GC content, BUSCO completeness, contamination/species confirmation result if available, and assembly accession or SRA run ID per isolate.

This is especially important because the authors discuss genotype–phenotype discordance and suggest that short-read assembly limitations may explain missing ESBL genes. Without assembly quality metrics, it is difficult to judge whether gene absence is biological or technical.

3. The AST section is too brief. The authors should specify the full antibiotic panel, disk potencies, interpretive criteria, quality control strains, incubation conditions, and whether intermediate isolates were consistently grouped with resistant isolates as “non-susceptible.” The tables state that percentages represent intermediate plus resistant isolates, but the methods should explicitly define this approach.

For Proteus mirabilis, the authors should also ensure that all antibiotics interpreted are appropriate for the species and clinical context. If intrinsic resistance was considered or excluded for any drug classes, this should be stated.

4. The manuscript states that ESBL detection was performed using the double-disk synergy test and carbapenemase production using mCIM. More detail is needed. For ESBL testing, the authors should specify which disks were used, distances between disks, the positive interpretation criteria, and QC strains. For carbapenemase testing, the authors should specify whether eCIM was performed. If eCIM was not done, the authors should avoid implying definitive carbapenemase class or mechanism.

The finding that 6 isolates were mCIM-positive but the genomic results do not clearly report carbapenemase genes needs further explanation. Were carbapenemase genes detected by AMRFinderPlus? If not, this discrepancy should be explicitly presented and discussed, including possible mechanisms such as porin alteration, ESBL/AmpC plus permeability changes, or limitations of gene detection.

5. The section on ESBL genotype–phenotype concordance is promising but incomplete. The authors report that among 20 phenotypically ESBL-positive sequenced isolates, 17 carried ESBL genes, and that five isolates carried ESBL genes but were phenotypically ESBL-negative. This should be presented as a 2 × 2 table with sensitivity, specificity, positive predictive value, negative predictive value, and overall agreement, with confidence intervals if possible.

The statement that there was “high concordance (85.0%)” is somewhat misleading because it only refers to ESBL-positive phenotypic isolates and does not account for the five genotype-positive/phenotype-negative isolates. The authors should calculate concordance across all 25 sequenced isolates and interpret discordance carefully.

6. The authors conclude that the data support endemic hospital circulation with partial clonal expansion and plasmid-mediated dissemination. However, the manuscript does not provide pairwise SNP distances, temporal distribution, ward admission overlap, patient movement data, or clear cluster definitions. The phylogeny alone, especially from 25 selected isolates, is insufficient to support strong transmission claims.

The authors should provide pairwise SNP distances within and between major ST clusters, define what constitutes a cluster, and include sampling dates if available. If such data are unavailable, the wording should be softened to indicate that the observed clustering is “consistent with” possible lineage expansion, rather than evidence of transmission.

7. The manuscript identifies IncQ1 and Col-type plasmid replicons and suggests plasmid-mediated dissemination. However, short-read replicon detection does not prove that ESBL genes are plasmid-located or transferred on those plasmids. The authors should avoid implying direct plasmid carriage of specific genes unless they provide contig-level co-localization, plasmid reconstruction, MOB-suite analysis, long-read sequencing, or conjugation evidence.

At minimum, the authors should report whether ESBL genes and replicon markers occurred on the same contigs or assembled plasmid bins. If not assessed, this should be stated as a limitation and the interpretation revised.

8. The manuscript uses Fisher’s exact or chi-square tests but does not always specify which test was used for each comparison. Given the small number of carbapenemase-positive isolates, Fisher’s exact test is appropriate. The authors should also consider multiple testing correction or clearly state that analyses are exploratory.

P-values alone are not enough. The authors should include effect sizes, such as odds ratios with confidence intervals, particularly for comparisons between ESBL-positive and ESBL-negative isolates and carbapenemase-positive and carbapenemase-negative isolates.

9. The current figures are difficult to read. Figure 2 and Figure 3 have small labels, crowded legends, and limited interpretability. The figure files embedded at the end of the manuscript appear cropped or low-resolution in places. For PLOS ONE, figures should be clear, self-contained, and readable without excessive zooming.

Figure 2 should be redesigned with larger labels, clearer legends, explicit annotation tracks, and a more informative caption explaining what each color track represents. Figure 3 should use a cleaner heatmap format with readable gene names and isolate labels. Figure 1 should also be improved, as the current plot contains limited information and could be replaced or supplemented by a clearer bar chart showing the distribution of resistance classes.

10. Several references appear problematic or mismatched. For example, some references cited for P. mirabilis virulence or resistance appear to concern other organisms or broader topics. The authors should carefully audit all references to ensure each citation supports the statement made. There are also formatting issues, including inconsistent spacing, duplicated Murray references, missing spaces before citations, and gene names not consistently formatted.

In addition, the manuscript contains several 2025 and 2026 references. The authors should confirm that all cited works are published, accessible, and correctly formatted with complete bibliographic details.

Minor comments

1. The abstract should clarify that WGS was performed only on a selected subset of 25 ESBL- and/or carbapenemase-associated isolates, not all 99 isolates.

2. The phrase “multidrug-resistance” should be corrected to “multidrug resistance” or “multidrug-resistant.”

3. Gene names should be consistently italicized where appropriate, and β-lactamase gene formatting should be standardized throughout.

4. The term “CRE+” in Table 3 should be reconsidered. Since the organism is Proteus mirabilis, and the phenotype refers to carbapenemase production, “carbapenemase-positive” or “mCIM-positive” may be clearer than “CRE+.”

5. The limitations section is useful but should be expanded to include the non-random WGS subset, lack of long-read sequencing, lack of plasmid-gene co-localization, lack of eCIM or carbapenemase gene confirmation, and limited epidemiological metadata.

Reviewer #2: The novelty of this study lies in its integrated genomic and phenotypic characterization of multidrug-resistant Proteus mirabilis isolates obtained from a military hospital environment, with particular emphasis on the diversity of ESBL determinants, plasmid-mediated dissemination, and population structure. Although antimicrobial resistance in P. mirabilis has been widely reported, comprehensive whole-genome epidemiological analyses from military healthcare settings remain limited.

The bioinformatic component appears to be the strongest aspect of the study; however, in my opinion, the overall novelty of the research is currently insufficient for publication in its present form. The study focuses primarily on a single area of investigation, which limits the broader scientific impact of the findings. Overall, the manuscript presents data mainly in an epidemiological context and offers limited scientific novelty.

**********

what does this mean?). If published, this will include your full peer review and any attached files.

If you choose “no”, your identity will remain anonymous but your review may still be made public.

**Do you want your identity to be public for this peer review?**  For information about this choice, including consent withdrawal, please see our Privacy Policy

Reviewer #1: No

Reviewer #2: No

**********

[NOTE: If reviewer comments were submitted as an attachment file, they will be attached to this email and accessible via the submission site. Please log into your account, locate the manuscript record, and check for the action link "View Attachments". If this link does not appear, there are no attachment files.]

To ensure your figures meet our technical requirements, please review our figure guidelines: https://journals.plos.org/plosone/s/figures

You may also use PLOS’s free figure tool, NAAS, to help you prepare publication quality figures: https://journals.plos.org/plosone/s/figures#loc-tools-for-figure-preparation.

NAAS will assess whether your figures meet our technical requirements by comparing each figure against our figure specifications.

# Author comment: Author response to Decision Letter 1

**DOI:** 10.1371/journal.pone.0355508.r002

13 Jun 2026

We thank the Editor and Reviewers for their careful evaluation of our manuscript and for their constructive comments. We have thoroughly revised the manuscript in response to all editorial and reviewer comments. Major revisions include clarification of the study methodology, expansion of genomic analyses, addition of supplementary information, revision of figures and tables, updates to the Funding and Competing Interests statements, and improvements to the presentation and interpretation of the results. A detailed point-by-point response to all comments is provided in the uploaded Response to Reviewers document.

Attachment Submitted filename: Reviewer 2 Response.docx

# Peer review: Decision Letter 1

**DOI:** 10.1371/journal.pone.0355508.r003

**Authors:** Zhi Ruan

8 Jul 2026

Dear Dr. Yusuf,

Thank you for submitting your manuscript to PLOS ONE. After careful consideration, we feel that it has merit but does not fully meet PLOS ONE’s publication criteria as it currently stands. Therefore, we invite you to submit a revised version of the manuscript that addresses the points raised during the review process.

Please submit your revised manuscript by Aug 22 2026 11:59PM. If you will need more time than this to complete your revisions, please reply to this message or contact the journal office at plosone@plos.org. When you're ready to submit your revision, log on to https://www.editorialmanager.com/pone/ and select the 'Submissions Needing Revision' folder to locate your manuscript file.

- A letter that responds to each point raised by the academic editor and reviewer(s). You should upload this letter as a separate file labeled 'Response to Reviewers'.
- A marked-up copy of your manuscript that highlights changes made to the original version. You should upload this as a separate file labeled 'Revised Manuscript with Track Changes'.
- An unmarked version of your revised paper without tracked changes. You should upload this as a separate file labeled 'Manuscript'.

If you would like to make changes to your financial disclosure, please include your updated statement in your cover letter. Guidelines for resubmitting your figure files are available below the reviewer comments at the end of this letter.

If applicable, we recommend that you deposit your laboratory protocols in protocols.io to enhance the reproducibility of your results. Protocols.io assigns your protocol its own identifier (DOI) so that it can be cited independently in the future. For instructions see: https://journals.plos.org/plosone/s/submission-guidelines#loc-laboratory-protocols. Additionally, PLOS ONE offers an option for publishing peer-reviewed Lab Protocol articles, which describe protocols hosted on protocols.io. Read more information on sharing protocols at https://plos.org/protocols?utm_medium=editorial-email&utm_source=authorletters&utm_campaign=protocols.

As the corresponding author, your ORCID iD is verified in the submission system and will appear in the published article. PLOS supports the use of ORCID, and we encourage all coauthors to register for an ORCID iD and use it as well. Please encourage your coauthors to verify their ORCID iD within the submission system before final acceptance, as unverified ORCID iDs will not appear in the published article. *Only*  the individual author can complete the verification step; PLOS staff *cannot*  verify ORCID iDs on behalf of authors.

We look forward to receiving your revised manuscript.

Kind regards,

Zhi Ruan, Ph.D.

Academic Editor

PLOS One

Journal Requirements:

If the reviewer comments include a recommendation to cite specific previously published works, please review and evaluate these publications to determine whether they are relevant and should be cited. There is no requirement to cite these works unless the editor has indicated otherwise.

[Note: HTML markup is below. Please do not edit.]

Reviewers' comments:

Reviewer's Responses to Questions

**Comments to the Author**

Reviewer #1: (No Response)

**********

2. Is the manuscript technically sound, and do the data support the conclusions?

Reviewer #1: Yes

**********

3. Has the statistical analysis been performed appropriately and rigorously?

Reviewer #1: N/A

**********

4. Have the authors made all data underlying the findings in their manuscript fully available?

The PLOS Data policy

Reviewer #1: Yes

**********

5. Is the manuscript presented in an intelligible fashion and written in standard English?

Reviewer #1: Yes

**********

Reviewer #1: The revised manuscript is substantially improved, and the authors have addressed several previous concerns. In particular, the manuscript now clarifies that whole-genome sequencing was performed on a selected subset of 25 isolates rather than all 99 isolates, expands the ESBL and carbapenemase phenotyping methods, adds some assembly-quality metrics, revises some transmission and plasmid-dissemination language, and expands the limitations section. These revisions strengthen the manuscript.

However, several important issues remain only partially addressed, and I recommend a further focused minor revision before acceptance.

First, the manuscript still does not provide a clear comparison between the 25 sequenced isolates and the full 99-isolate collection. Because the WGS subset was purposively selected and enriched for ESBL- and/or carbapenemase-associated phenotypes, the authors should add a supplementary table comparing the sequenced subset with the full collection by specimen type, ward/source, ESBL status, carbapenemase status, MDR profile, and key phenotypic resistance patterns.

Second, the sequencing-quality information remains incomplete. The added assembly metrics are useful, but Supplementary Table S2 should also include total reads, coverage depth, isolate-level SRA run IDs or assembly accessions, and species confirmation/contamination assessment if available. These details are important because the manuscript discusses genotype–phenotype discordance, and readers need enough information to assess whether gene absence may be biological or technical.

Third, the ESBL genotype–phenotype concordance analysis has not been adequately addressed. The previous review requested a 2 × 2 table with sensitivity, specificity, positive predictive value, negative predictive value, and overall agreement. The revised manuscript provides a narrative description but does not provide this table. Based on the numbers reported, 20 sequenced isolates were phenotypically ESBL-positive, 17 of these carried ESBL-associated genes, and five phenotypically ESBL-negative isolates carried ESBL-associated genes. Therefore, the overall agreement appears to be 17/25, or 68%, and should not be described as high or substantial without qualification. This analysis should be clearly presented and interpreted cautiously.

Fourth, carbapenemase genotype–phenotype interpretation remains underdeveloped. The authors clarified that mCIM was performed and that eCIM was not used, which is helpful. However, the manuscript should clearly state whether carbapenemase genes were detected among the six mCIM-positive isolates. If no carbapenemase genes were detected, this should be explicitly discussed as phenotype–genotype discordance. If genes were detected, they should be reported isolate by isolate.

Fifth, the antimicrobial susceptibility testing methods still require more complete reproducibility details. The Methods should list the full antibiotic panel, disk potencies, interpretive criteria, and whether any antibiotics were excluded or interpreted cautiously because of intrinsic resistance or species-specific considerations for Proteus mirabilis.

Sixth, some interpretation of phylogeny and hospital circulation remains too strong. Without pairwise SNP distances, sampling dates, patient movement data, ward overlap, or defined genomic clusters, the authors should avoid wording that suggests proven endemic circulation or transmission. Wording such as “is consistent with possible circulation” or “suggests possible lineage expansion” would be more appropriate.

Seventh, plasmid interpretation should remain limited to replicon detection. The authors correctly acknowledge that short-read data cannot confirm plasmid structure or gene–plasmid co-localization. Any remaining wording implying plasmid-mediated dissemination of specific ESBL genes should be removed unless supported by contig-level co-localization, plasmid reconstruction, MOB-suite analysis, long-read sequencing, or conjugation data.

Eighth, the statistical analysis remains limited. The authors now state that Fisher’s exact test was used and that comparisons involving carbapenemase-positive isolates are exploratory, which is appropriate. However, p-values from very small groups should not be overemphasized. Where feasible, odds ratios with confidence intervals should be added for the main comparisons, or the authors should explicitly state that effect-size estimates were not reported because of instability due to sparse cells.

Ninth, the figures remain a concern. Although the authors state that the figures were improved, the submitted versions still appear cropped or difficult to read, especially the phylogeny and virulence heatmap. Figure 2 should be regenerated with readable isolate labels, clear metadata tracks, larger legends, and a caption explaining all colour annotations. Figure 3 should be presented as a clean heatmap with legible gene names and isolate labels. Final figure files should be checked at publication size.

Tenth, the references and formatting still need careful checking. Some citations appear mismatched to the statements they support. For example, a statement on P. mirabilis flagellar systems and virulence appears to cite a reference concerning E. coli CTX-M-27 plasmids, which does not support that claim. The authors should re-audit all references, especially those cited for P. mirabilis virulence, plasmids, ESBL epidemiology, and recent 2025/2026 claims.

Finally, the manuscript requires a careful final language and formatting edit. There are still missing spaces, inconsistent section numbering, and awkward phrases such as “extensive multidrug-resistant associated.” The plasmid section numbering also appears inconsistent. These issues should be corrected before acceptance.

The manuscript contains valuable data and is potentially suitable for publication after a focused minor revision. The remaining issues do not require major new experiments, but they do require clearer reporting, improved figures, more cautious interpretation, completion of requested supplementary information, and correction of residual citation and formatting problems.

**********

what does this mean?). If published, this will include your full peer review and any attached files.

If you choose “no”, your identity will remain anonymous but your review may still be made public.

**Do you want your identity to be public for this peer review?**  For information about this choice, including consent withdrawal, please see our Privacy Policy

Reviewer #1: No

**********

[NOTE: If reviewer comments were submitted as an attachment file, they will be attached to this email and accessible via the submission site. Please log into your account, locate the manuscript record, and check for the action link "View Attachments". If this link does not appear, there are no attachment files.]

To ensure your figures meet our technical requirements, please review our figure guidelines: https://journals.plos.org/plosone/s/figures

You may also use PLOS’s free figure tool, NAAS, to help you prepare publication quality figures: https://journals.plos.org/plosone/s/figures#loc-tools-for-figure-preparation.

NAAS will assess whether your figures meet our technical requirements by comparing each figure against our figure specifications.

# Author comment: Author response to Decision Letter 2

**DOI:** 10.1371/journal.pone.0355508.r004

19 Jul 2026

Dear Editor,

Thank you for the opportunity to revise our manuscript. We have carefully considered all comments from the Academic Editor and reviewers and have revised the manuscript accordingly. A detailed, point-by-point response to each comment is provided in the uploaded Response to Reviewers document. All changes made in the revised manuscript have been highlighted where appropriate.

We believe that these revisions have substantially improved the manuscript and hope it is now suitable for publication in PLOS ONE.

Thank you for your time and consideration.

Sincerely,

Yacob Mohammed Yusuf

On behalf of all authors

Attachment Submitted filename: Reviewer Response.docx

# Editor report: Decision Letter 2

**DOI:** 10.1371/journal.pone.0355508.r005

**Authors:** Zhi Ruan

22 Jul 2026

Genomic Epidemiology of Multidrug-Resistant and ESBL-Producing Proteus mirabilis in a Military Referral Hospital in Ethiopia

PONE-D-26-15733R2

Dear Dr. Yusuf,

We’re pleased to inform you that your manuscript has been judged scientifically suitable for publication and will be formally accepted for publication once it meets all outstanding technical requirements.

Within one week, you’ll receive an e-mail detailing the required amendments. When these have been addressed, you’ll receive a formal acceptance letter and your manuscript will be scheduled for publication.

An invoice will be generated when your article is formally accepted. Please note, if your institution has a publishing partnership with PLOS and your article meets the relevant criteria, all or part of your publication costs will be covered. Please make sure your user information is up-to-date by logging into Editorial Manager at Editorial Manager® and clicking the ‘Update My Information' link at the top of the page. For questions related to billing, please contact billing support.

If your institution or institutions have a press office, please notify them about your upcoming paper to help maximize its impact. If they’ll be preparing press materials, please inform our press team as soon as possible -- no later than 48 hours after receiving the formal acceptance. Your manuscript will remain under strict press embargo until 2 pm Eastern Time on the date of publication. For more information, please contact onepress@plos.org.

Kind regards,

Zhi Ruan, Ph.D.

Academic Editor

PLOS One

Additional Editor Comments (optional):

Reviewers' comments:
